# A MYCN-driven de-differentiation profile identifies a subgroup of aggressive retinoblastoma

**DOI:** 10.1038/s42003-024-06596-6

**Published:** 2024-07-30

**Authors:** Tatsiana Ryl, Elena Afanasyeva, Till Hartmann, Melanie Schwermer, Markus Schneider, Christopher Schröder, Maren Wagemanns, Arthur Bister, Deniz Kanber, Laura Steenpass, Kathrin Schramm, Barbara Jones, David T. W. Jones, Eva Biewald, Kathy Astrahantseff, Helmut Hanenberg, Sven Rahmann, Dietmar R. Lohmann, Alexander Schramm, Petra Ketteler

**Affiliations:** 1 Department of Pediatric Hematology and Oncology, University Hospital Essen, Essen, Germany; 2 Algorithms for Reproducible Bioinformatics, Genome Informatics, Institute of Human Genetics, University Hospital Essen, University of Duisburg-Essen, Essen, Germany; 3 Institute of Human Genetics, University Hospital Essen, University Duisburg Essen, Essen, Germany; 4 Human and Animal Cell Lines, Leibniz Institute DSMZ German Collection of Microorganisms and Cell Cultures, 38124 Braunschweig, Germany; 5 Division of Pediatric Glioma Research, Hopp Children’s Cancer Center (KiTZ), Heidelberg, Germany; 6 National Center for Tumor Diseases (NCT), NCT Heidelberg, a partnership between DKFZ and Heidelberg University Hospital, Heidelberg, Germany; 7 German Cancer Research Center (DKFZ), Heidelberg, Germany; 8 Department of Pediatric Hematology and Oncology, Heidelberg University Hospital, University of Heidelberg, Heidelberg, Germany; 9 Department of Ophthalmology, Medical Faculty, University of Duisburg-Essen, 45147 Essen, Germany; 10 Department of Pediatric Oncology and Hematology, Charité – University Medicine Berlin, Berlin, Germany; 11 Algorithmic Bioinformatics, Center for Bioinformatics Saar and Saarland University, Saarland Informatics Campus, Saarbrücken, Germany; 12 Laboratory for Molecular Oncology, Department of Medical Oncology, West German Cancer Center, University Hospital Essen, University of Duisburg-Essen, Essen, Germany

**Keywords:** Paediatric cancer, Cancer epigenetics, Eye cancer

## Abstract

Retinoblastoma are childhood eye tumors arising from retinal precursor cells. Two distinct retinoblastoma subtypes with different clinical behavior have been described based on gene expression and methylation profiling. Using consensus clustering of DNA methylation analysis from 61 retinoblastomas, we identify a MYCN-driven cluster of subtype 2 retinoblastomas characterized by DNA hypomethylation and high expression of genes involved in protein synthesis. Subtype 2 retinoblastomas outside the MYCN-driven cluster are characterized by high expression of genes from mesodermal development, including *NKX2-5*. Knockdown of *MYCN* expression in retinoblastoma cell models causes growth arrest and reactivates a subtype 1-specific photoreceptor signature. These molecular changes suggest that removing the driving force of MYCN oncogenic activity rescues molecular circuitry driving subtype 1 biology. The MYCN-RB gene signature generated from the cell models better identifies MYCN-driven retinoblastoma than *MYCN* amplification and can identify cases that may benefit from MYCN-targeted therapy. MYCN drives tumor progression in a molecularly defined retinoblastoma subgroup, and inhibiting MYCN activity could restore a more differentiated and less aggressive tumor biology.

## Abstract (web summary)

DNA methylation analysis identifies a MYCN-driven subgroup of aggressive retinoblastoma that may benefit from *MYCN*-targeted therapy.

## Introduction

Retinoblastomas are retinal tumors originating from postmitotic cone photoreceptor precursor cells^1–3^. Most retinoblastomas are characterized by a biallelic inactivation of *RB1*, the RB transcriptional corepressor 1, and ~45% of patients with retinoblastoma carry a constitutional, pathogenic *RB1* variant^4^. Early diagnosis of intraocular retinoblastoma leads to high survival rates in high-income countries, while metastatic retinoblastoma is rare because the natural boundaries of the eye prevent metastatic spread in early disease stages^5^. In low and middle income country, metastatic spread of retinoblastoma is more common and leads to significantly lower survival rates^5^. Intraocular retinoblastoma can be treated with enucleation of the eye or, especially in patients with bilateral disease, with a range of therapies to preserve the eye globe and vision. Biopsies of intraocular retinoblastoma are obsolete, so that samples from early disease that received eye-preserving therapy are scarce. Advances in and increasing use of eye-preserving therapies complicate retinoblastoma molecular genetic characterization and development of innovative targeted therapies, although there is hope that this may be overcome by serial liquid biopsies in the future^6^.

Recurrent genetic alterations other than *RB1* are rare in retinoblastoma^7–10^, while copy number aberrations of chromosomes 1, 6, and 16 are characteristic^11^. DNA methylation-based clustering combined with gene expression profiling have distinguished two retinoblastoma subtypes with differences in clinical characteristics, genomic alterations, and, potentially, prognoses^12–14^. Subtype 1 retinoblastomas (equivalent to cluster 2 in Kooi et al.^13^) are characterized by few genetic and chromosomal alterations, mostly limited to *RB1* loss and 6p gain, and show gene expression resembling signatures of maturing photoreceptor cells that Kooi et al. termed a “photoreceptorness signature”^13^ and Liu et al. termed a “cone marker signature”^12^. Subtype 2 retinoblastomas (equivalent to cluster 1 described by Kooi^13^) are genetically more heterogeneous, carry more genetic and chromosomal alterations than subtype 1 retinoblastomas and express high levels of neuronal markers and genes for stemness with low levels of photoreceptor-related genes^12^. High TFF1 expression has been described as a surrogate marker for subtype 2 retinoblastoma^12,15^. Preliminary clinical data suggest that subtype 2 retinoblastomas are more aggressive and at higher risk for metastasis^12,15^ and, consequently, may require higher treatment intensity. To tailor treatment accordingly in the future, further characterization of the molecular and biological characteristics of each subtype is warranted.

*MYCN* oncogene amplification has been observed in a small subgroup of retinoblastomas grouped into subtype 2 retinoblastoma^12,13,16^. The MYC protein family are basic helix-loop-helix (bHLH) transcription factors regulating proliferation, differentiation, cell-cycle progression, protein synthesis, metabolism, and apoptosis^17–21^. Amplifications of *MYCN* or *MYC* occur, respectively, in the childhood cancers, neuroblastoma, and medulloblastoma, and the resulting protein overexpression is associated with aggressive growth and poor clinical outcome^22–24^. In retinoblastoma, *MYCN* amplification (*MYCN*^*A*^) occurs in *RB1*^−/−^*MYCN*^*A*^ retinoblastomas and the rare, RB1-proficient *MYCN*^*A*^ retinoblastomas. RB1*-*proficient *MYCN*^*A*^ retinoblastomas are currently considered a separate disease entity with very aggressive behavior, in which MYCN is thought to exclusively drive tumorigenesis^25,26^. RB1-proficient *MYCN*^*A*^ retinoblastoma could potentially arise from a different cell of origin or at a different maturation stage than *RB1*^−/−^ retinoblastomas.

Here we aimed to define the impact of MYCN activity in the context of retinoblastoma subtypes 1 and 2 in a cohort of 61 retinoblastoma samples enriched by six RB1-proficient *MYCN*^*A*^ and two extraocular metastatic relapse samples. Genome-wide DNA methylation profiling was used to molecularly classify the 61 primary retinoblastomas. We sought circuitry defining the molecular phenotypes through bioinformatics analyses on RNA sequencing datasets from 52 samples. Our aim was to precisely define the molecular patterns of retinoblastoma with oncogenic MYCN activity within subtype 2. We generated *MYCN*-knockdown retinoblastoma cell models to assess functional effects and define a MYCN-RB signature, as a potential application to identify patients with retinoblastomas driven by oncogenic MYCN activity who could benefit from MYCN-directed treatment.

## Results

### A distinct hypomethylated DNA pattern characterizes MYCN-driven retinoblastomas

**Fig. 1:**
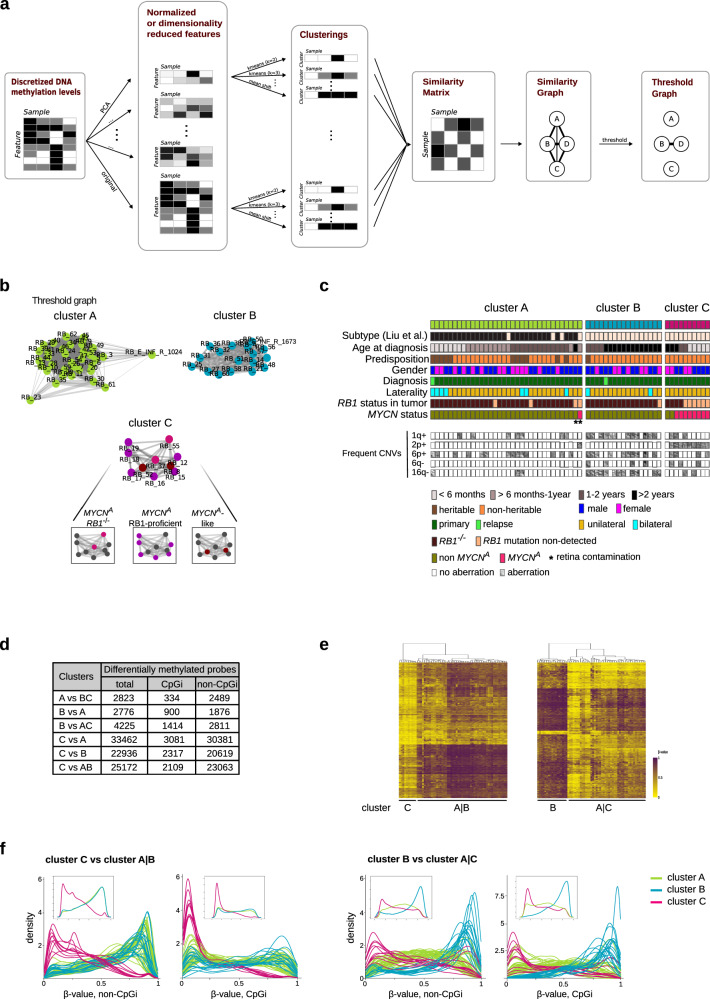
MYCN-driven retinoblastomas have distinct molecular (epi)genetic and clinical features. **a** Flowchart displaying method of DNA methylation data clustering. The flowchart summarizes the steps performed to build a threshold graph reflecting consensus clustering of the DNA methylation data from 61 retinoblastomas. **b** Separation of 3 retinoblastoma clusters by global DNA methylation-based consensus clustering (62 datasets of retinoblastomas), cluster A (*n* = 35, green), cluster B (*n* = 17, turquoise) and cluster C (*n* = 10, magenta). MYCN-driven cluster retinoblastoma included *RB1*^−/−^*MYCN*^*A*^ (pink, *n* = 2), RB1-proficient *MYCN*^*A*^ (purple, *n* = 6), and *RB1*^−/−^non-*MYCN*^*A*^ (maroon, *n* = 2). **c** How molecular tumor and clinical characteristics in the 61 patients with retinoblastoma differed in between the 3 clusters and correlation of clustering with the 2 subtypes grouped by the 8-CpG classifier^12^ (Supplementary Data 1). **d** The numbers of differentially methylated CpG sites (Welsh t-test; abs.diff 0.2; Benjamini–Hochberg adjusted *p* < 0.001) in and outside of CpG islands (CpGi) are listed. **e** Differentially methylated CpGs in cluster C compared to cluster A|B and in cluster B compared to cluster A|C are depicted in the 2 heatmaps. **f** Density plots for DNA methylation levels (ß-values) of differentially methylated probes in cluster C vs. cluster A|B (left) and cluster B vs. cluster A|C (right). Inlet plots represent median density plots per cluster.

We examined epigenetic and genomic levels for a detailed molecular view of subtypes 1 and 2 in a cohort of 61 primary retinoblastoma samples enriched by 6 RB1-proficient *MYCN*^*A*^ retinoblastoma samples (4 previously described^25^) and 2 samples of relapsed, extraocular retinoblastomas (Supplementary Data 1, 2). *RB1* status was derived from routine diagnostic sequencing and *MYCN* amplifications were derived from global DNA methylation profiles using whole-genome Illumina 450k or EPIC (850k) arrays (61 samples). We applied an unsupervised DNA methylation-based cluster assembly and increased the robustness of this clustering using a beta-value discretization approach^27^ in a consensus clustering of 8857 unsupervised clusterings (k-means and agglomerative or hierarchical), affinity propagation, mean-shift, DBSCAN, and spectral clusterings, each with various sets of hyperparameters (Fig. 1a). In contrast to the previously described 2 retinoblastoma subtypes, our consensus clustering distinguished 3 retinoblastoma groups: cluster A (*n* = 35, included 2 datasets from 1 sample), cluster B (*n* = 17) and cluster C (*n* = 10, Fig. 1b). The Liu et al. 9-CpG classifier^12^ designed for 450k methylation array data (and based on 8 probes also used in the EPIC array) showed a correspondence between cluster A and subtype 1 retinoblastoma, and between clusters B and C and subtype 2 retinoblastoma (Fig. 1c, Supplementary Fig. S1a). In line with this, the CpG subsets defined by Li et al.^28^ for each cluster corresponded with our clusters (Supplementary Fig. S1b).

More CpGs were differentially methylated between clusters C and A (33462 probes) than between clusters B and A (2776 probes, Fig. 1d), emphasizing a distinct methylation pattern in cluster C retinoblastomas. Hypomethylation was characteristic for differentially methylated CpGs in cluster C (Fig. 1e), while most differentially methylated CpGs in cluster B retinoblastomas were hypermethylated (Fig. 1e, Supplementary Fig. S1c). Cluster C-specific hypomethylation and cluster B-specific hypermethylation occurred both inside and outside CpG islands (Fig. 1f). Only 8.4% of CpGs differentially methylated in cluster C were within islands, while 33.5% of differentially methylated CpGs in cluster B were in CpG islands. All *MYCN*^A^ retinoblastomas (6 RB1-proficient *MYCN*^*A*^, 2 *RB1*^*−/−*^*MYCN*^*A*^) clustered together with 2 *RB1*^−/−^retinoblastomas lacking *MYCN* amplifications as cluster C (Fig. 1c). The 2 retinoblastomas lacking *MYCN* amplifications had aberrations that could trigger oncogenic MYCN activity through other routes. The focal 13q31.3 amplification in 1 retinoblastoma harbored *MIR17HG*, a microRNA known to activate MYC(N) signaling, and part of *GPC5* (Supplementary Fig. S2a, c, Supplementary Data 3). The other retinoblastoma had multiple genomic abnormalities on chromosomes 6, 7, and 8, in line with chromothripsis, chromoanasynthesis or chromoplexy including an amplification containing the downstream MYC(N) signaling component, *BRAF* (Supplementary Fig. S2b, c, Supplementary Data 3).

Children with cluster A retinoblastomas were significantly younger at diagnosis (median: 0.87 years) than patients with cluster B retinoblastomas (median: 2.36 years, *p*-value (*p*)_Wilcoxon rank test_ = 5.785e−08; Fig. 1c, Supplementary Fig. S2d, Supplementary Data 1). The age at which children were diagnosed with *RB1*^−/−^ retinoblastomas in cluster C (median: 2.86 years) did not statistically differ from children with cluster B retinoblastomas (median: 2.36 years, *p*_Wilcoxon rank Test_ = 0.83; Supplementary Fig. S2d), while the 6 patients with RB1-proficient retinoblastomas in cluster C were significantly younger (median: 0.38 years, vs cluster B *p*_Wilcoxon rank test_ = 0.006961; Supplementary Fig. S2d). Classification separated the 2 extraocular relapsed retinoblastomas, grouping 1 in cluster A (subtype 1) and the other in cluster B (subtype 2). Gain of 6p was observed in all 3 clusters, while 1q gain and 16q loss were observed predominantly in cluster B, and 2p gain was exclusive to cluster C (Fig. 1c, Supplementary Fig. S2e). Genetic mutations (whole-exome sequencing) other than *RB1* were rare, apart from somatic mutations in *BCOR* and the *ARID* family genes, *ARID1A* and *ARID4A*, as previously reported^7,12^ (Supplementary Data 4, 5). Consensus clustering subdivided subtype 2 retinoblastomas into 2 clusters, B and C, with distinct patterns in *MYCN* amplification, genomic rearrangements, and methylation profiles.

### *NKX2-5* and *MYCN* are the transcription factors best demarcating clusters B and C

**Fig. 2:**
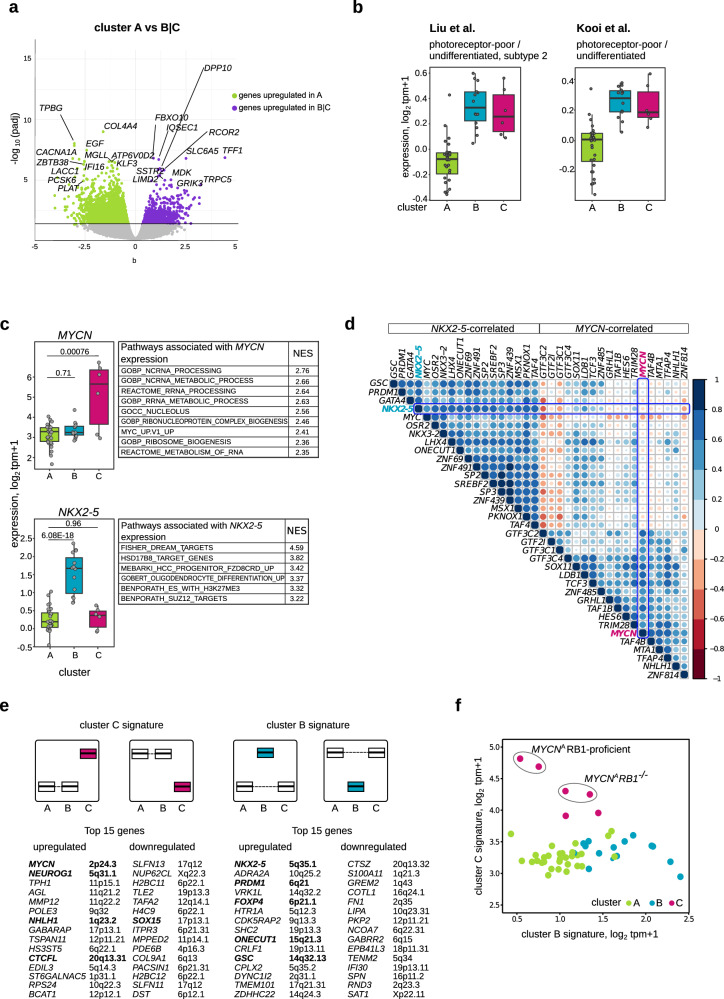
Transcriptomic landscape in clusters B and C retinoblastomas (including RB1-proficient retinoblastoma). **a** Volcano plot of differentially expressed transcripts between cluster A and cluster B|C. Up- and downregulated transcripts are plotted in violet and green, respectively. Key transcripts are plotted with the corresponding annotated gene symbols (Supplementary Data 6). **b** Box plots showing distribution of expression scores from genes representative of photoreceptor-poor, undifferentiated retinoblastoma (Liu score^12^: 3105 genes positively expressed; 3088 genes downregulated, adjusted *p* ≤ 0.05; or Kooi-score^13^: 3425 genes upregulated; 3477 genes downregulated, adjusted *p* ≤ 0.05) in 52 retinoblastomas. **c** Box plots showing aggregated log1p-transformed transcript tpm (transcripts per kilobase million) values and combined Storey-Tibshirani-adjusted *p* for *MYCN* and *NKX2-5* expression (left) and associated gene sets (right) in 52 retinoblastomas. **d** Expression of transcription factors correlating with *NKX2-5* and *MYCN* is illustrated in the circle plot. Color and size of the circles represent the correlation coefficients. **e** Schemes showing expression of genes defined for cluster B and C gene signatures. The top 15 upregulated and downregulated genes within these signatures are listed with the corresponding cytobands (complete outputs, Supplementary Data 15, 16). Genes encoding transcription factors are in bold. **f** Scatter plot demonstrating the differentially expression of genes from cluster B (524 transcripts, x-axis) and cluster C (117 transcripts, y-axis) signatures in 52 retinoblastomas. The average of log1p-transformed tpm values for a gene set was used as a signature score of each retinoblastoma sample. Boxes show upper and lower quantile and median. Whiskers extend from the hinge to ±1.5 times the interquartile range.

We applied RNA sequencing to the 52 samples from the 61-retinoblastoma cohort (Supplementary Data 2). To characterize the activated signaling pathways in each cluster, we compared the RNA sequencing datasets from the 52 primary retinoblastomas cluster-wise (31 cluster A, 15 cluster B, 6 cluster C retinoblastomas) using *sleuth*^29^. Comparing gene expression from retinoblastomas in cluster A (subtype 1) with clusters B|C (subtype 2) retrieved 4887 differentially expressed transcripts corresponding to 3109 protein-coding genes (2403 upregulated genes in cluster A, 706 upregulated genes in clusters B|C, |b|> 0.3; Fig. 2a, Supplementary Data 6). Gene-set enrichment analysis (GSEA) showed that more strongly expressed genes in cluster A retinoblastomas were involved in inflammatory and interferon-gamma responses (Supplementary Fig. S3a, Supplementary Data 7). Clusters B and C shared high expression of genes from signatures defined for photoreceptor-poor, undifferentiated retinoblastoma (Liu score^12^: *p*AvsB = 3.1e−09, *p*AvsC = 2.2e−05, *p*CvsB = 0.78; Kooi Score^13^: *p*AvsB = 1.3e−08, *p*AvsC = 4.6e−05, *p*CvsB = 0.78; Wilcoxon rank-sum test; Fig. 2b). In line with this, pairwise comparisons of clusters B and C with cluster A (Supplementary Data 8, 9) showed that the majority of upregulated genes in cluster B (73%) and cluster C (82%) belonged to genes previously shown to be representative of photoreceptor-poor, undifferentiated retinoblastoma^12,13^ (Supplementary Fig. S3b). Clusters B and C shared 41 upregulated genes and 113 downregulated genes that included *TFF1, EBF3, BCL11A,* and *SOX11* (Supplementary Data 10, Supplementary Fig. S3c). High-level *MYCN* expression characterized cluster C, while strong expression of the *NKX2-5* and *GATA4* transcription factors characterized cluster B (Fig. 2c, Supplementary Fig. S3d, Supplementary Data 8, 9). However, transcription factors co-expressed with *MYCN* or *NKX2-5* (Supplementary Data 11) were not mutually exclusive. Only *MYC* expression showed a trend to anti-correlate with *MYCN* itself and factors co-expressed with *MYCN* (Fig. 2d).

High MYC(N) target gene expression characterized cluster C, as well as heightened gene activity for ribosomal function and protein synthesis and low expression of genes involved in cilia function (GSEA, Supplementary Data 12). Cluster B was characterized by low immune-related gene activity and high target expression for a component of the RB-like, E2F and multi-vulval class B (DREAM) complex, hydroxysteroid dehydrogenase and polycomb repressive complex 2 (PRC2, Supplementary Data 13). Compared to cluster C, a subset of immune response-specific signatures was depleted and genes involved in transcriptional regulation were overrepresented in cluster B (Supplementary Fig. S3d, Supplementary Data 14). These data implicate distinct pathways in clusters C and B, with both routes leading to retinoblastoma de-differentiation.

We selected differentially expressed gene signatures defining either cluster C or B to better understand differences in cluster expression circuitry. A step-wise statistical filtering approach was applied to genes differentially expressed among clusters A through C to obtain signatures specific to cluster B or C. The signature defining cluster C contained fewer differentially expressed transcripts (cluster C: 372, cluster B: 1886, Supplementary Data 15, 16). Among the top 15 genes strongly expressed in only cluster C were several encoding bHLH transcriptions factors, including *MYCN*, *NHLH1*, and *NEUROG1*, while the developmentally regulated homeobox transcription factors, *NKX2-5*, *ONECUT1*, and *GSC* were among the genes defining cluster B (Fig. 2e). No other transcription factor was more abundant (transcript level) than *MYCN* in *MYCN*^*A*^ retinoblastomas. The signature defining cluster C is expressed at a low level in cluster B (Fig. 2f, Supplementary Fig. S3e), and vice versa. GSEA recapitulated the expression signatures in pairwise comparisons of clusters B and C against A. Notably, retinoblastomas in both clusters B and C strongly expressed genes from a stemness signature and genes associated with neuroblasts and neuronal progenitors. Retinoblastomas in clusters B and C appear to share transcriptional characteristics, but be driven by distinct molecular circuitry indicated by their specific transcriptional hallmarks.

### CpGs differentially methylated in clusters B and C are localized to genes defining these clusters

**Fig. 3:**
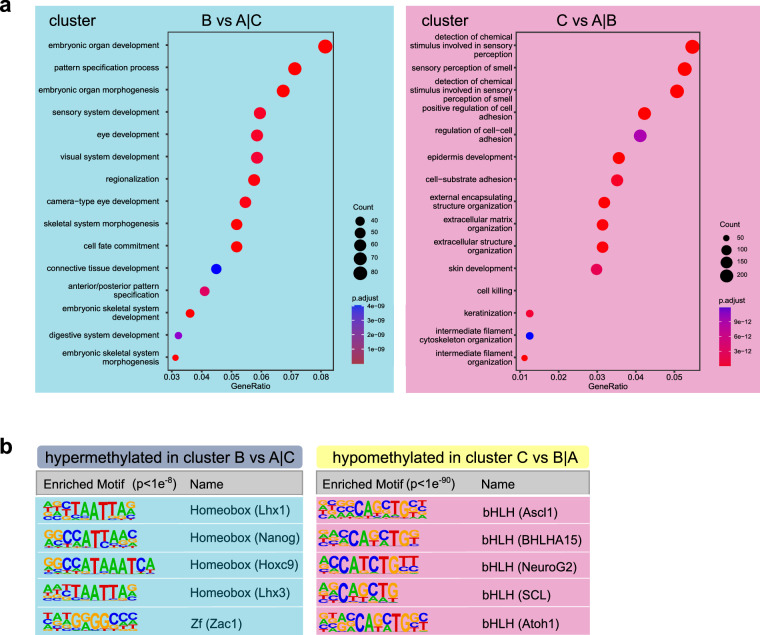
Differentially methylated CpGs in retinoblastoma clusters B and C. **a** Biological processes retrieved from gene ontology enrichment analysis of the genes matching to differentially methylated CpGs between clusters B and A|C (left) and differentially methylated CpGs between clusters C and A|B (right). Dot size and color represent the number of genes and enrichment significance, respectively. x-axis indicates the gene enrichment ratio (GeneRatio) of a biological process GO term. **b** Selected motifs identified in 50-bp sequences surrounding CpGs that are hypermethylated in clusters B vs. A|C (4109 sequences, left) or hypomethylated in clusters C vs. A|B (25073 sequences, right) identified by HOMER known DNA-motif enrichment (complete outputs, Supplementary Data 17, 18).

We detected hypermethylation in cluster B and hypomethylation in cluster C retinoblastomas (Fig. 1e, f). Differentially methylated genes in cluster B were 97% hypermethylated (compared to A│C) and in cluster C were 99% hypomethylated (compared to A│B). The presence of specific transcriptional hallmarks of clusters B and C suggested a correlative connection between our DNA methylation-based clustering and defining molecular circuitry in the retinoblastomas. To explore processes that DNA methylation could regulate, we analyzed enrichment of gene ontology (GO) terms corresponding to differentially methylated CpGs in each cluster. Hypermethylated genes in cluster B were involved in camera-eye development, regulating neuronal function and synapse organization (Fig. 3a). Cluster C was enriched with hypomethylated genes involved in smell perception (Fig. 3a, Supplementary Fig. S4a). Minimal regions around hypermethylated CpGs, distinguishing cluster B, were enriched for binding motifs specific for homeodomain and zinc-finger transcription factors (HOMER analysis, Fig. 3b, Supplementary Data 17). In contrast, the E-box (bHLH-binding motifs) variants, CACCTG and CATCTG, were enriched in minimal regions around hypomethylated CpGs in cluster C (HOMER analysis, Fig. 3b, Supplementary Data 18), indicating a potentially more open chromatin configuration for MYCN-driven gene regulation. Our results suggest that bHLH factors, such as MYCN, are highly expressed in cluster C retinoblastoma.

**Fig. 4:**
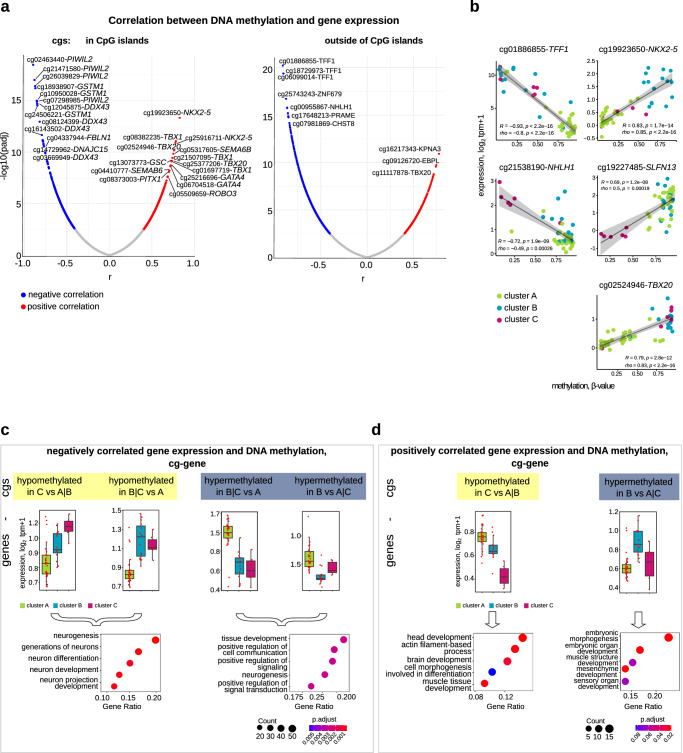
Integrative analysis of DNA methylation and gene expression in retinoblastoma clusters B and C. **a** Volcano plot for Pearson correlations between mRNAs and methylation values of corresponding local CpGs in CpG island (left) and non-CpG island (right) contexts in 52 retinoblastomas (Supplementary Data 19, 20). Y-axes represent logarithm of *p*-value of each correlation coefficient. X-axes represent the Pearson correlation coefficient, r. Selected highly correlated CpG-mRNA pairs are labeled in blue (negative correlation) and red (positive correlation). **b** Relationship between gene expression in log1p-transformed tpm values and DNA methylation of CpGs (β-values) for selected genes in 52 primary retinoblastomas. Y-axes represent mRNA expression. X-axes represent DNA methylation. Each dot corresponds to a retinoblastoma sample and is color-coded by retinoblastoma cluster (green, cluster A; *turquoise*, cluster B; magenta, cluster C). Pearson (R) and Spearman (rho) coefficients with corresponding *p*-values, linear regression line and confidence interval are indicated for each comparison. Box plots showing average of log1p-transformed tpm values of negatively (**c**) and positively correlated (**d**) genes in retinoblastoma clusters (upper panels) and dot plots showing biological processes associated with these genes (lower panels). Boxes show upper and lower quantile and median. Whiskers extend from the hinge to ±1.5 times the interquartile range or the highest/lowest value.

**Fig. 5:**
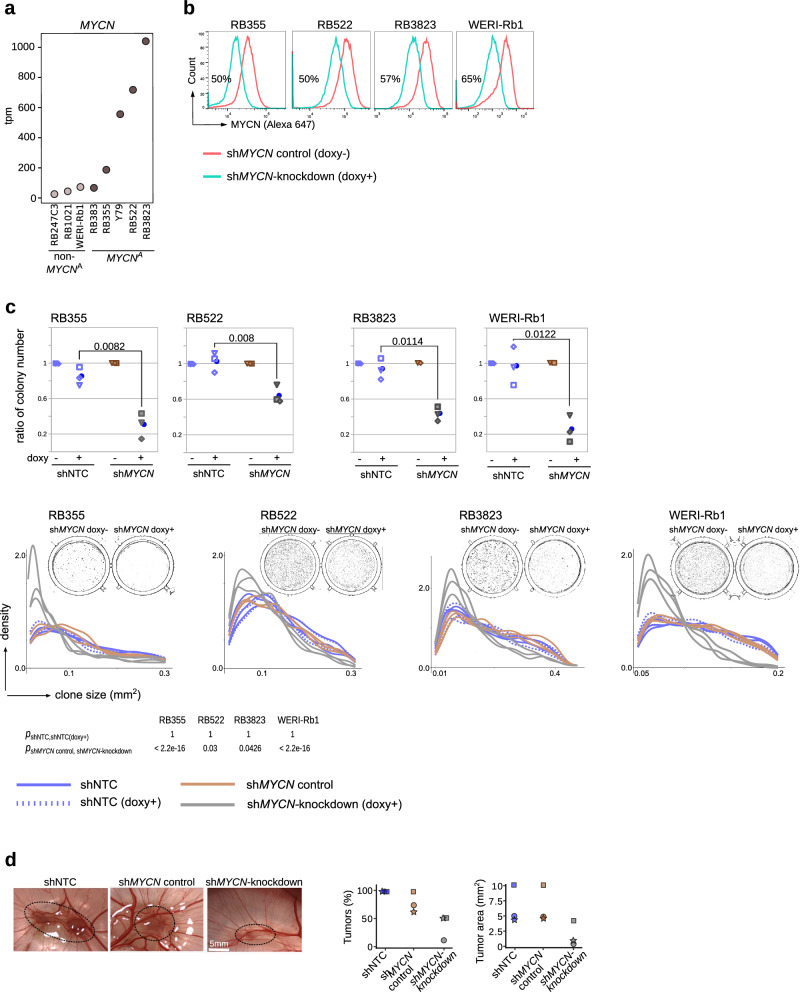
*MYCN* knockdown reduces clonogenicity and tumor formation in *MYCN*-knockdown retinoblastoma cell models. **a** *MYCN* mRNA expression in retinoblastoma cell lines determined by RNAseq. **b** Flow cytometry analysis of MYCN protein expression in RB355, RB522, RB3823, and WERI-Rb1 cell lines transduced with doxycycline-inducible lentiviral sh*MYCN* expression vector. MYCN protein was measured 48 h after adding doxycycline to the media. The results represent 3 experiments. **c** Soft agar colony formation analysis in retinoblastoma cell lines. Plots (upper panel) show ratios of colony numbers in shNTC-expressing (doxycycline-treated cells vs non-treated cells) and sh*MYCN*-expressing (doxycycline-treated cells vs non-treated cells) retinoblastoma cell lines from three independent experiments. Data are from 3 independent experiments, with average values (blue bots) indicated and *p*-values calculated with Welch t-test reported. Density plots (lower panel) for colony size in shNTC-expressing and sh*MYCN*-expressing retinoblastoma cell lines. Each line corresponds to one technical replicate. Representative images from sh*MYCN*-expressing non-treated and doxycycline-treated cells are shown for each graph (shNTC-expressing non-treated and doxycycline-treated cells are in Supplementary Fig. S6d). Combined *p*-values calculated with one-sided Wilcoxon rank-sum test for the arbitrary difference in clone size (10% of maximum clone size) are shown. **d** Effects of *MYCN* knockdown in RB355 cells and the chick chorioallantoic model. Photos and plots, respectively, show the tumors in eggs at developmental stage E17 and the fraction of tumor cells and tumor area in engrafted eggs at stage E17. The results represent 3 replicates with 10 eggs for each condition.

We integrated our RNA sequencing and DNA methylation data to explore local regulation of gene expression via autosomal CpGs (in and outside islands) annotated to the protein-coding genes in each signature (Fig. 4a). The proposed surrogate marker for subtype 2 retinoblastoma, *TFF1*, was hypomethylated and strongly expressed in clusters B and C (Fig. 4a, b). The bHLH transcription factor, *NHLH1*, was hypomethylated and strongly expressed in cluster C (Fig. 4a, b). The majority of genes with differentially methylated CpGs (6320 CpGs of 2465 genes) negatively correlated with expression (R ≤ 0.4, Fig. 5a), while 2261 CpGs of 992 genes, including *NKX2-5*, positively correlated with gene expression (R ≥ 0.4; Fig. 4a, b, Supplementary Data 19–23). GO enrichment analysis revealed enrichment of neuronal development and function for negatively correlated hypomethylated genes in clusters B and C (Fig. 4c). Negatively correlated hypermethylated genes in clusters B and C were enriched for cell-to-cell communication and neuronal development terms (Fig. 4c). Positively correlated genes in cluster B and cluster C were enriched for the GO terms, muscle tissue development, mesenchyme development and mesenchymal cell differentiation (Fig. 4d). Our comparisons of differentially methylated CpGs between clusters (Fig. 1d) also identified 28% of CpGs analyzed in correlation analysis. Genes associated with enhancer elements previously annotated in developing retina or retinoblastoma^30,31^ were hypomethylated and highly expressed in our cohort (Supplementary Data 24, Supplementary Fig. S4b). Yet, enhancer elements, with the exception of hypomethylated in cluster C enhancers associated with *MYCN* and *NHLH1*, lacked cluster-specific differences in methylation (Supplementary Data 24). Our methylation and expression data confirm the previous association of *TFF1* with subtype 2 retinoblastoma, provide further support for the defining role of MYCN activity in cluster C.

### *MYCN* knockdown rescues molecular circuitry driving subtype I in retinoblastoma cell models

We profiled 9 previously described retinoblastoma cell lines^32^ (Supplementary Fig. S5a, Supplementary Data 2) with our multi-omics approach. The RB1-proficient cell lines, RB522 and RB3823, and the *RB1*^*−/−*^*MYCN*^*A*^ cell line, Y79, expressed the highest *MYCN* mRNA and MYCN protein levels and showed the highest proliferation rates (Fig. 5a, Supplementary Fig. S5b). Applying the Liu et al. 9-CpG classifier^12^ to these data grouped all 9 cell lines with subtype 2 retinoblastoma (Supplementary Fig. S5c). The 4 *MYCN*^*A*^ cell lines comprised a separate branch closer to cluster C than B based on hierarchical clustering of mRNA expression of transcription factors expressed in these 2 retinoblastoma clusters (Supplementary Fig. S5d, e). The highest expression scores for cluster C signature genes were detected in the fast-growing, *MYCN*^*A*^ RB3832, RB522 and Y79 cell lines (Supplementary Fig. S5f). Since no cell lines are derived from subtype 1 retinoblastoma, we explored the functional effects of MYCN in the subtype 2 background by generating *MYCN* knockdown models from these cell lines.

**Fig. 6:**
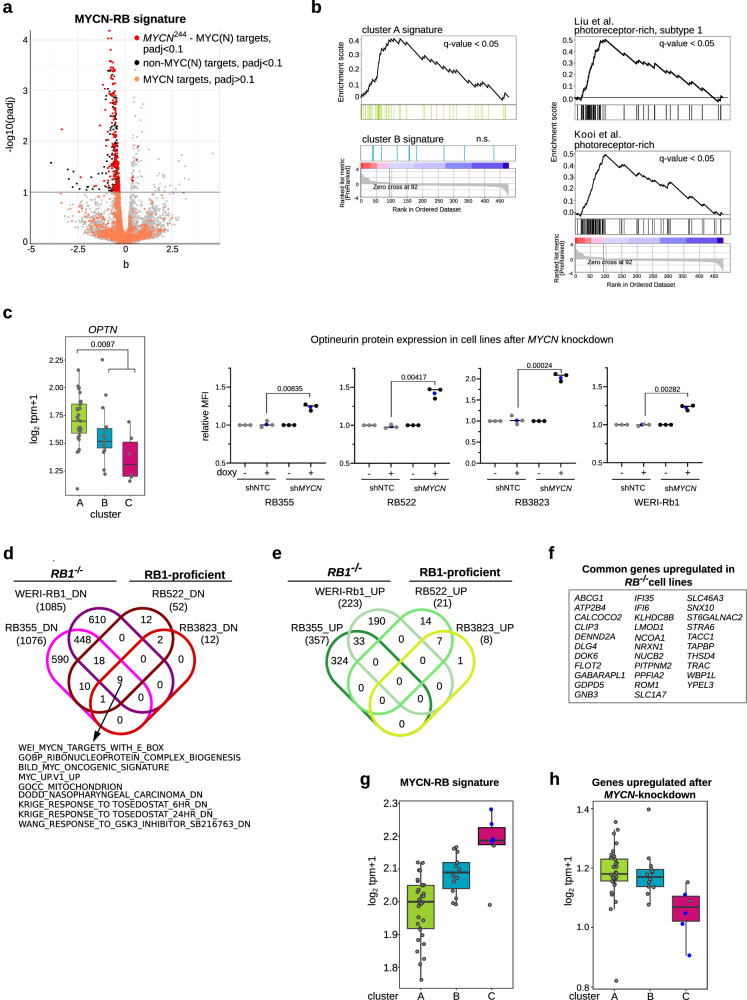
Gene re-expression representative of photoreceptor-rich retinoblastoma in *MYCN*-knockdown cell models. **a** Volcano plot shows the MYCN-RB signature of 244 genes derived from the overlap of previously identified MYCN targets and *sleuth*-modeled *MYCN*-knockdown expression pattern (Supplementary Data 25). Red dots correspond to significantly regulated MYCN targets (Storey-Tibshirani adjusted *p* ≤ 0.1). Orange dots correspond to other MYCN targets (Storey-Tibshirani adjusted *p* > 0.1). Black dots represent non-MYCN targets significantly downregulated in *sleuth*-modeled *MYCN*-knockdown expression pattern. **b** GSEA showing genes overexpressed in cluster A, genes representative of photoreceptor-rich retinoblastoma^12,13^ and the cluster B signature in the *sleuth*-modeled *MYCN*-knockdown expression pattern. Y-axis indicates enrichment score (ES). X-axis shows pathway genes. The dual-colored band represents the degree of correlation in the expression of these genes with *MYCN*-knockdown (red, *MYCN*-knockdown; blue, control). **c** Box plot (left) showing aggregated log1p-transformed transcript tpm values for *OPTN* expression and combined Storey-Tibshirani-adjusted *p* in 52 retinoblastomas. OPTN protein expression (right) in *MYCN*-knockdown retinoblastoma cell lines 48 h after doxycycline treatment. Dot plots show ratios of median fluorescence intensity (MFI) for OPTN in shNTC-expressing (doxycycline-treated cells vs non-treated cells) and sh*MYCN*-expressing (doxycycline-treated cells vs non-treated cells) retinoblastoma from three independent experiments. Data are from 3 independent experiments, with average values (blue bots) indicated and *p*-values calculated with Welch t-test reported. Representative plots of flow cytometry for OPTN are shown in Supplementary Fig. S7c. Venn diagrams showing the pathways (FDR adjusted *p* ≤ 0.05) associated with downregulated genes (**d**) and upregulated genes (**e**) in *MYCN*-knockdown retinoblastoma cell lines. The 9 pathways depleted in all 4 cell lines are listed. **f** List of genes upregulated in both *RB1*^−/−^ cell lines. Box plots showing the expression scores of the MYCN-RB signatures (**g**), and upregulated genes (**h**) after *MYCN*-knockdown in each of the 3 retinoblastoma clusters. Boxes show upper and lower quantile and median. Whiskers extend from the hinge to ±1.5 times the interquartile range.

To define molecular and functional changes driven by MYCN in subtype 2 retinoblastomas, inducible knockdown models were generated from 4 retinoblastoma cells lines. We selected the 2 RB1 proficient *MYCN*^A^ cell lines (shRNA-based *MYCN* knockdown in RB522, RB3832) because of high MYCN RNA and protein expression and two RB1^−/−^ cell lines with and without *MYCN* amplification (shRNA-based *MYCN* knockdown in RB355 *MYCN*^*A*^ cell lines and the *MYCN*-diploid WERI-Rb1 cell line). Knockdown reduced MYCN levels by 50–65% in cell models (Fig. 5b, Supplementary Fig. S6a). The proportion of cycling cells and the ability to form colonies was reduced in culture (Supplementary Fig. S6b, c, Fig. 5c, Supplementary Fig. S6d, e) and tumor growth was reduced in the in vivo chorioallantoic membrane model (Fig. 5d). Analysis of RNA sequencing data across all 4 cell models identified a common set of 477 genes that were differentially expressed (93 upregulated, 385 downregulated, Storey-Tibshirani *p* ≤ 0.1; Supplementary Data 25). More than 50% of commonly downregulated genes (*n* = 244) were known MYC(N) targets^23,33–35^ and define a MYCN-RB signature (Fig. 6a, Supplementary Data 26). *MYCN* knockdown downregulated expression of genes involved in protein synthesis and cell metabolism (Supplementary Data 27, second most common functional group).

Genes known to be markers for photoreceptor cells (*NRL*, *OPTN*, *SLC17A7, PLEKHB1, GNGT2, ATP2B1*, and *BAZ2B*) were among those upregulated in models after *MYCN* knockdown (Supplementary Data 25). MYCN knockdown upregulated genes descriptive of the photoreceptor-rich cluster A retinoblastoma (Fig. 6b) and downregulated genes representative of the photoreceptor-poor, undifferentiated and cluster C retinoblastoma (Supplementary Fig. S7a, b) in cell models. None of the genes defining cluster B were regulated by *MYCN* knockdown (*sleuth*-modeled *MYCN*-knockdown expression profile, Fig. 6b; pairwise non-induced/induced cell model comparisons, Supplementary Data 28), indicating MYCN plays no role in the molecular circuitry driving cluster B retinoblastoma. We confirmed upregulation of cluster A expressed *OPTN* on protein level using flow cytometry analysis (Fig. 6c, Supplementary Fig. S7c). Notably, pathways associated with genes downregulated by *MYCN* knockdown were consistent across all 4 cell models (Fig. 6d). The pathways associated with upregulated genes were similar within each of the 2 *RB1*^−/−^ cell models (RB355 and WERI-Rb1) and RB1-proficient cell models (RB522 and RB3832), but differed between the 2 groups (Fig. 6e). In the absence of functional RB1, *MYCN* knockdown upregulated more genes encoding photoreceptor or ciliary proteins (compared with RB1-proficient cell lines; Fig. 6f, Supplementary Data 29), suggesting a stronger redifferentiation of the cells.

We applied the MYCN-RB signature to our RNA sequencing dataset from 52 primary retinoblastomas. MYCN-RB expression scores were lowest in cluster A and highest in cluster C (*pAC* = 0.00058, *pAB* = 0.00016, *pBC* = 0.02; Fig. 6g), peaking in *MYCN*^*A*^ retinoblastomas (*pMYCN*^*non-A*^*-MYCN*^*A*^_Wilcoxon rank sum test_ = 3.694e−06) and correlating with *MYCN* expression (R = 0.69, R^*MYCNnon-A*^ = 0.60, *p* < 0.00001). We also assessed the opposite picture of expression (genes upregulated by *MYCN* knockdown) from our *sleuth*-modeled *MYCN*-knockdown expression profile in the 52 primary retinoblastomas. Retinoblastomas lacking *MYCN* amplifications strongly expressed this gene group (*pMYCN*^*non-A*^*-MYCN*^*A*^_Wilcoxon rank sum test_ = 0.0002623), and expression was highest in cluster A, followed by cluster B (pAC = 0.00058, pBC = 0.00227, pAB = 0.37503; Wilcoxon rank-sum test; Fig. 6h). In our cohort of primary retinoblastoma, the MYCN-RB signature generated in the cell line model was able to identify MYCN-driven retinoblastomas with and without *MYCN*^*A*^, which cluster separately based on methylation as cluster C retinoblastomas. To validate this finding, we reanalyzed the DNA methylation array data of the Liu et al. cohort^12^ using our subsets of cluster B- and cluster C-specific CpGs, which revealed two cluster C retinoblastomas (Supplementary Fig. S8a, b). Based on Liu et al. RNA expression data, these two cluster C retinoblastoma and one retinoblastoma without methylation data showed the highest MYCN-RB signature scores (Supplementary Fig. S8c). These three tumors were identified as *MYCN*^*A*^, supporting the validity of MYCN-RB signature to identify *MYCN*-amplified retinoblastomas.

Likewise, the expression of genes descriptive of the photoreceptor-rich retinoblastoma in the cell line models after *MYCN* knockdown shows that MYCN is involved in maintaining undifferentiated state of retinoblastoma cells.

## Discussion

Here, we first define a MYCN-driven group of subtype 2 retinoblastoma characterized by hypomethylation and high expression of MYCN target genes. This *subtype 2-MYCN* (cluster C) comprises not only the rare RB1-proficient *MYCN*^*A*^ retinoblastoma but also *RB1*^−/−^ retinoblastomas with overactive components of MYCN signaling. Knockdown of *MYCN* in cell models reactivated the differentiated subtype 1 photoreceptor signature, suggesting a transition between the differentiated subtype 1 retinoblastoma and the more aggressive subtype 2 retinoblastoma that can potentially be reversed via targeting MYCN. We also defined and validated a MYCN-RB gene signature to identify retinoblastomas that could benefit from MYCN-targeting treatment.

**Fig. 7:**
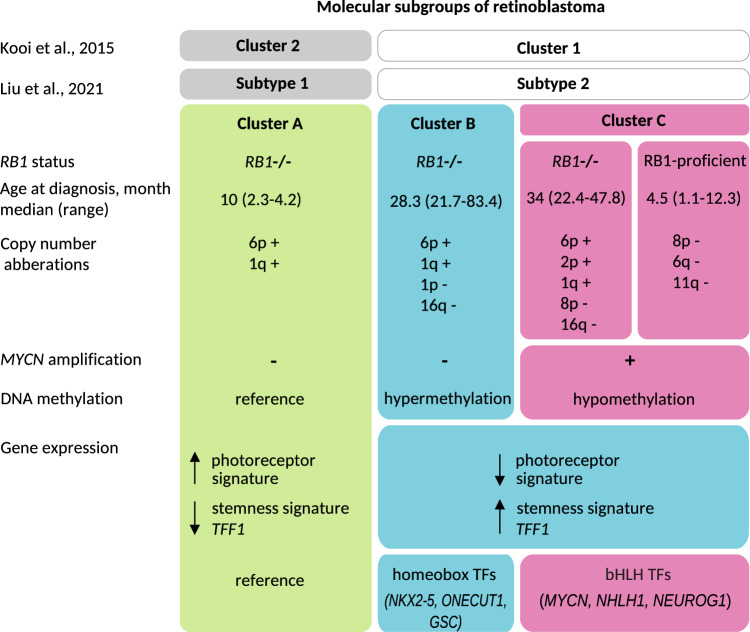
Overview of characteristics of 3 retinoblastoma clusters (including RB1-proficient retinoblastoma).

In contrast to the previously described hypermethylation pattern^12,16^ of subtype 2 retinoblastoma, this *subtype 2-MYCN* (cluster C) was characterized by hypomethylation and showed distinct patterns of genomic rearrangements, gene expression profiles and clinical characteristics to cluster B, underlining biological differences between these two clusters within subtype 2 (Fig. 7). Our data identify cluster C as a subgroup with different defining molecular circuitry and hallmarks in subtype 2 retinoblastomas^12^. Yet, the absence of anti-correlated gene expression between cluster B- and cluster C-specific transcription factors indicate that expression programs in cluster B and C retinoblastomas are probably not mutually exclusive. However, correlation of gene expression and methylation clearly confirmed *MYCN* as the transcription factor best demarcating cluster C from B.

The MYCN-driven cluster C included not only *MYCN*-amplified retinoblastomas, but two samples with high MYCN target expression that lacked *MYCN* amplifications, suggesting alternative ways of activating MYCN-driven tumor progression in retinoblastoma. Gene amplification is one of several mechanisms that lead to *MYCN* deregulation and activation of MYCN targets^36^. Several microRNAs, including *MIR17HG*, are involved in the complex regulation of MYC(N) signaling^37^, so that it is plausible that *MIR17HG* amplification drives MYCN signaling in the selected cluster C retinoblastoma. The high expression of *MYC* and other bHLH transcription factors (*NHLH1* and *NEUROG1*) was similar among cluster C retinoblastomas, regardless of *MYCN* amplification status. The enrichment of CACCTG and CATCTG E-box variants, which are the preferred by TWIST, NEURO, and TCF/LEF family transcription factors and can bind MYCN under deregulated conditions^38^, emphasize the importance of bHLH transcription factors for cluster C retinoblastomas. Remarkably, hypomethylation of several olfactory receptor genes was characteristic for *subtype 2-MYCN* (cluster C). The olfactory receptor gene family is the largest in the human genome^39^. Ectopically expressed olfactory receptor genes have been linked with differentiation of cancer cells and prognosis in various cancers^40^. Activation of olfactory receptor genes may reflect de-differentiation of subtype 2-MYCN (cluster C) retinoblastoma. As *MYCN* amplification alone was insufficient to identify MYCN-driven retinoblastomas, we defined the MYCN-RB signature using data from *MYCN*-knockdown cell models. The MYCN-RB signature identified cluster C retinoblastomas and can be used to identify retinoblastoma cases that may benefit from MYCN-directed therapies.

*MYCN* is a known oncogene in various embryonal tumors and *MYCN* amplification is a well-known prognostic factor for patients with neuroblastoma^41^. A previous study demonstrated that MYCN oncogenic programs differed between retinoblastoma and neuroblastoma^42^. In contrast to neuroblastoma^43^, the *MYCN*-associated methylation pattern was characterized by hypomethylation in our retinoblastoma cohort, but results from our *MYCN*-knockdown cell models support a significant overlap between MYCN targets in retinoblastoma and known MYCN and MYC targets. Genes involved in protein synthesis appeared to be controlled by MYCN in retinoblastoma, consistent with previous findings in other cancers^44,45^. This supports the idea of MYCN cooperation with translation processes in retinoblastoma and opens up perspectives for new drug targets in MYCN-driven retinoblastomas. In retinoblastoma, *MYCN* amplification occurs in both the more common *RB1*^−/−^ and rare RB1-proficient backgrounds^25,46^. RB1-proficient retinoblastoma has been described as a separate retinoblastoma entity with very aggressive behavior, in which MYCN is thought to be the exclusive driver of tumorigenesis^25,46^. Our consensus clustering based on genome-wide methylation did not distinguish between RB1-proficient and *RB1*^−/−^ *MYCN*-amplified retinoblastomas, as other groups have reported^16^. While this is a relatively large group of the exceedingly rare RB1-proficient retinoblastomas, the comparison is limited by small cohort size, such that batch effects cannot completely be excluded. Apart from the previously reported very young age of diagnosis for RB1-proficient retinoblastoma, we did not observe differences in gene expression, chromosomal or genetic features between the *RB1*^−/−^ and RB1-proficient *MYCN*-amplified retinoblastomas. Only in our cell models did differences become apparent after *MYCN* knockdown, when *RB1*^−/−^ cell lines showed more pronounced expression of the photoreceptor signature than RB1-proficient retinoblastomas. DNA methylation and gene expression profiling in further RB1-proficient *MYCN*-amplified retinoblastomas will help to better characterize differences in molecular circuitry in *RB1*^−/−^ and RB1-proficient *MYCN*-amplified retinoblastoma.

*MYCN* knockdown in our cell models caused a partial growth arrest and reactivated the photoreceptor gene signature of subtype 1 retinoblastoma in the subtype 2 cell lines. These observations can be explained by differentiation of retinoblastoma cells along a cone photoreceptor cell trajectory, and can be interpreted as a dynamic connection between subtype 2 and subtype 1 retinoblastoma. MYCN blocks differentiation pathways and maintains pluripotency during development and cancer pathogenesis^47–49^. Enforced reduction of *MYCN* expression in neuroblastoma cells is also associated with cell differentiation^50^. Kapatai et al.^14^ has suggested subtype 2 retinoblastoma originates from an earlier retinal lineage or an early uncommitted cell type, but subtype 2 retinoblastoma could alternatively originate from subtype 1 retinoblastoma via acquiring additional genetic and chromosomal alterations^3,13^. All available retinoblastoma cell lines grouped as subtype 2 retinoblastoma. This could either reflect that the more differentiated subtype 1 retinoblastoma is not amenable to culturing or that subtype 1 retinoblastoma acquire additional genetic and epigenetic alterations in culture and advance to subtype 2 retinoblastoma. The differentiation observed upon *MYCN* knockdown could hint that subtype 1 retinoblastoma is able to transition to *subtype 2-MYCN* (cluster C) retinoblastoma upon *MYCN* activation.

The lack of serial tissue sampling during retinoblastoma treatment leaves the question whether subtype 1 transitions into subtype 2 retinoblastoma by acquiring additional mutations^3,13^ or whether both subtypes arise independently from the same progenitor cell at different stages of maturation unresolved^12,14^. Subtype 2 occurs in older children, shows a more undifferentiated gene expression profile and predominates in metastatic cases^12^. One of the two extraocular retinoblastomas that we analyzed was unexpectedly a cluster A, subtype 1 retinoblastoma without elevated TFF1 expression. Remarkably, this patient is a long-term survivor despite localized central nervous system metastasis.

Few extraocular cases have been subtyped because molecular genetic analysis is conducted almost exclusively on cases treated in high-income countries, where nearly all retinoblastomas are diagnosed and treated during early intraocular stages. Accordingly, most primary retinoblastomas in our cohort were subtype 1 retinoblastomas (35/61, 57%). Countries with limited resources have high mortality rates from retinoblastoma^5^ because cases most often progress to extraocular metastasis. This implies that either geographic factors affect molecular retinoblastoma subtype distribution or that subtype 1 retinoblastoma can advance to subtype 2 retinoblastoma or take an aggressive clinical course given time. Collaborative international research projects are needed to answer this question and better define molecular retinoblastoma subtypes.

Given its correlation with rapid tumor progression and poor prognosis in several embryonal cancers MYCN is considered an ideal therapeutic target, but many direct or indirect MYCN modulators failed to result in an efficient MYCN specific therapy^51,52^. New approaches including histone deacetylases inhibitors, Aurora Kinase-A Inhibitors and Bromodomain and extra-terminal domain family Inhibitors show promising interaction with MYCN and its pathways^51^. As biopsies are obsolete in retinoblastoma, all tissue samples in our study derived from patients with advanced intraocular disease treated with enucleation. Therefore, molecular genetic characteristics of small intraocular retinoblastoma remain undetermined. This potential bias may be overcome in the future by the use of liquid biopsies. Results of molecular genetic characterization of cfDNA derived from aqueous humor at diagnosis, during treatmentis promising to provide information on smaller retinoblastoma, serial samples and correlation with response to therapy^53^. Analysis of cfRNA from aqueous humor could in the future also allow to identify MYCN-RB signature in retinoblastoma treated with eye-preserving therapy.

Our data suggest that de-differentiation from subtype 1 to *subtype 2-MYCN* (cluster C) retinoblastoma can be reversed by inhibiting MYCN. The newly defined MYCN-RB signature supports identification of patients who may benefit from MYCN-directed therapies, and presents a biomarker-guided option for patient selection in clinical trials incorporating agents targeting MYCN. Our data may also be applicable to *MYCN* signaling pathways in other cancers in which *RB1* inactivation occurs, including lung, ovarian and breast cancers.

## Methods

### Patients and samples

We selected a cohort of 61 intraocular primary retinoblastoma samples from patients diagnosed and treated between 2007 and 2014 at the University Hospital Essen (written informed consent for use in research available). We enriched our cohort for the rare retinoblastoma types, RB1-proficient *MYCN*^*A*^ (DNA and RNA from 4 retinoblastomas that were first described^25^ was reanalyzed) and extraocular metastatic relapses (patient and tumor characteristics in Supplementary Data 1). The remaining 2 RB1-proficient *MYCN*^*A*^ samples lacked detected *RB1* variants in routine diagnostics, raising the suspicion this case is RB1-proficient *MYCN*^*A*^. The data from the 2 patients with relapsed extraocular retinoblastoma evaluated in this study was produced and kindly provided by the INFORM program^54–57^. Selection criteria for all 59 primary retinoblastoma samples were: (1) consent for research projects available, (2) adequate DNA purity and quality, (3) no treatment prior to enucleation and (4) availability of sufficient tumor and blood DNA. Samples were obtained after primary enucleation or resection of the metastasis, directly frozen and stored at −80 °C until DNA and RNA preparation. *RB1* genotyping was performed within genetic testing on matched blood and tumor samples at the request of individuals or their legal guardians with the aim to identify oncogenic alterations in *RB1*.

### Cell lines

WERI-Rb1 (RRID:CVCL_1792) and Y79 (RRID:CVCL_1893) cell lines were purchased from the German Collection of Microorganisms and Cell Cultures (DSMZ, Braunschweig, Germany). RB1021 (RRID:CVCL_S624), RB247C (RRID:CVCL_2704), RB355 (RRID:CVCL_S611), RB383 (RRID:CVCL_S626), RB3823 (RRID:CVCL_ZF07), RB522 (RRID:CVCL_ZF04) were kindly provided by Brenda Gallie (Department of Ophthalmology and Vision Sciences, Hospital for Sick Children, Toronto). Rbl30 (RRID:CVCL_S621) was kindly provided by Ralf Küppers (University Hospital Essen). Cell line identity was validated in-house (Institute for Human Genetics) using *RB1* mutational status and short tandem repeats. All retinoblastoma cell lines are well established, and were previously molecularly, cellularly and functionally characterized^32^. Cell lines were cultivated in Dulbecco’s modified Eagle medium (ThermoFisher Scientific) supplemented with 15% fetal calf serum (Thermo Fisher Scientific), 11 µM β-mercaptoethanol (MilliporeSigma), and 100 U/ml penicillin/streptomycin (Thermo Fisher Scientific) in a humidified atmosphere with 5% CO_2_ at 37 °C. All cell lines except RB355 (adherent monolayer) were cultivated in suspension. WERI-RB1, RB247C3, and RB1021 are *RB1*^*−/−*^ cell lines lacking *MYCN* amplifications. Y79, Rb383 and RB355 are *RB1*^*−/−*^*MYCN*^*A*^ cell lines. RB3823 and RB522 are RB1-proficient *MYCN*^*A*^ cell lines with functional RB1.

### Generating doxycycline-inducible MYCN-knockdown cell models

*MYCN* knockdown was achieved using the Dharmacon doxycycline-inducible lentiviral SMARTvector system with PGK promoter, TurboGFP and 3 human *MYCN* knockdown target sequences (CGAGCTGATCCTCAAACGA targeting 3′-UTR and open reading frame, ACGTCCGCTCAAGAGTGTC targeting 3′-UTR and open reading frame and CCACATAAGGGGTTTGCCA targeting only the 3′-UTR). The SMARTvector Inducible lentiviral NTC PGK-TurboGFP served as non-targeting control. The *MYCN*-knockdown vector targeting only the 3′-UTR performed best in all cell lines, and was used to generate all models. The polyethylenimine (PEI) method was used to create and transduce Vesicular Stomatitis Virus Glycoprotein-G (VSV-G)-pseudotyped replication-deficient lentiviral particles into HEK293T cells as described previously^58^. Briefly, 6 µg each of envelope plasmid (pczVSV-G), helper plasmid (pCD/NL-BH) and pSMART plasmids were mixed with 45 µg PEI and incubated with HEK293T cells. After 24 h, medium was changed to Iscove’s modefied Dulbecco’s medium (MilliporeSigma) supplemented with 10% FCS, 1% penicillin/streptomycin. Lentiviral supernatant was harvested at 24 h, filtered (0.45 µm filters) and added (1:2 dilution) to retinoblastoma cell cultures with protamine phosphate (final concentration 5 µg/mL medium, Millipore Sigma). Medium was aspirated off cells after 24 h, and cells washed once with phosphate-buffered saline (PBS, ThermoFisher Scientific) before culturing in complete medium for 7 days. Transduced cells were selected with 1 µg/mL puromycin (ThermoFisher Scientific). Cells were cultured in medium containing 0.5 µg/mL doxycycline (STEMCELL Technologies) for 24–72 h to establish a *MYCN*-knockdown population prior to further analysis.

### Protein staining for flow cytometry

MYCN protein staining was performed using AlexaFluor 647-labeled antibodies against MYCN (sc-53993, Santa Cruz Biotechnology) and mouse IgG2a (sc-24637, Santa Cruz Biotechnology). OPTN protein staining was performed using non-conjugated antibodies (#70928, Cell Signaling) and secondary Alexa Fluor 647-labeled antibody (A-21244, Rabbit IgG (H + L) Cross-Adsorbed Secondary Antibody, Thermo Fisher Scientific, and Rabbit mAb IgG XP®Isotype Control (#2975 Cell Signaling). Cells (10^6^) were prefixed with 4% paraformaldehyde (Morphisto) for 15 min at room temperature, resuspended in ice-cold methanol and fixed overnight at −20 °C. For immunostaining, cells were washed in PBS, incubated for 30 min in 1% bovine serum albumin (Roth) and 0.1% Triton X-100 in PBS (MilliporeSigma) then incubated 1 h at room temperature with the antibody (0.5 mg per sample). Then washed and if required incubated for 1 h with secondary antibody (dilution 1:400).

### Assaying cell proliferation and viability

The proportion of cells in S phase was determined using EdU incorporation and cell cycle analysis. Cells were incubated with 10 µM EdU (Lumiprobe) for 1 h prior to collection, then 10^6^ cells fixed in 4% paraformaldehyde (Morphisto) at room temperature for 15 min and in 90% ice-cold methanol before storing at −20 °C. Fixed cells were permeabilized for 30 min in 1 M Tris buffer (pH 7.4) containing 1% bovine serum albumin and 0.1% Triton X-100, then stained for 30 min with 3 µM Cy5 azide (Lumiprobe) in 1 M Tris (pH 7.4) with 2 mM CuSO_4_ and 20 mg/ml ascorbic acid. Cells were subsequently washed with PBS and counterstained with DAPI (Millipore, Sigma) for flow cytometric cell cycle analysis. Flow cytometry was conducted on a Beckman Coulter CytoFLEX instrument. Data analysis used FlowJo software V10 (Becton Dickinson). Viability was assessed in retinoblastoma cell lines and models using the 3-(4,5-dimethylthiazol-2-yl)-2,5-diphenyltetrazolium bromide (MTT) assay (Roth) according to manufacturer’s instructions. Cells (10^5^ cells for suspension cell lines, 5 × 10^4^ for adherent cells) were seeded onto 96-well plates in quadruplicate for MTT assay, and if *MYCN* knockdown was to be induced, incubated 24 h before 0.5 µg/ml doxycycline treatment. For soft agar assays, cells were trypsinised, and 0.5 × 10^4^ cells were resuspended in a top agar solution containing 0.35% agar, then poured onto a base layer containing 0.7% agar in 12-well plates. Each well was covered with 0.7 ml of media. If *MYCN* knockdown was to be induced, cells were supplemented with 0.5 µg/ml doxycycline in top agar solution and upper media. Plates were incubated under standard culture conditions for 2 weeks. Colonies were fixed and stained with 10% methanol, 10% acetic acid and 0.1% of crystal violet. For clonogenicity assays, cells (0.5 × 10^4^) were seeded onto 12-well plates pre-coated with poly-L-lysine (Thermo Fisher Scientific). *MYCN* knockdown was induced by adding doxycycline to a final concentration of 0.5 µg/ml, and plates were incubated under standard culture conditions for 2 weeks. Cells were fixed in 75% methanol, 25% acetic acid and stained with 10% methanol, 10% acetic acid, and 0.5% of crystal violet. The colony counting, calculation of colony areas was performed using Ilastik^59^-processed segmented images and ImageJ software (Rasband, WS, ImageJ, U.S. National Institutes of Health, Bethesda, MD, USA). Proliferation was assessed in the *MYCN*-knockdown RB355 and RB355 cell models using the chick chorioallantoic membrane (CAM) assay. Knockdown was induced in model cells (0.5 µg/ml doxycycline) 48 h before engrafting. Induced or uninduced model cells (10^6^/50 µl PBS) were grafted onto the chorioallantoic membrane of E10 stage chick embryos after cutting a window into the eggshell. Proliferation was assessed 7 days after engraftment, at stage E17, by enlarging the windows to photograph the entire anterior chorioallantoic membrane region and carefully extract the tumors. Tumors were weighed to assess volumetric growth, and tumor areas were assessed using ImageJ software. The results represent 3 replicates with 10 eggs per test condition.

### Genetic, epigenetic, and genomic analyses

*RB1* variants were identified using DNA from fresh-frozen tumor samples or DNA from blood and one or more of the following in routine diagnostics (Dept. of Human Genetics, Essen) as previously described^60–63^ analysis of allele loss in tumors, cytogenetic analysis, denaturing high performance liquid chromatography, exon-by-exon sequencing, multiplex ligation-dependent probe amplification, methylation-sensitive PCR, quantitative fluorescent multiplexed PCR, quantitative real-time PCR, real-time PCR and single-strand conformation polymorphism analysis. *RB1* mutational mosaicism was diagnosed when the signal ratio for the mutant:normal allele in blood DNA < 50%, but detecS§. If available, DNA from an additional tissue source (i.e., buccal mucosa) was analyzed for *RB1* mosaic cases. Genome-wide methylation was analyzed using the Illumina Infinium Human Methylation 450k array (21 retinoblastoma samples) or EPIC 850k BeadChip array (39 retinoblastoma samples). Sample RB_33 was analyzed on both arrays to control for batch effects (Supplementary Data 1). Whole-exome sequencing (WES) was performed in 37 retinoblastomas (Supplementary Data 1).

### RNA sequencing

RNAseq analysis was performed in 52 of the 61 intraocular retinoblastomas (Supplementary Data 2). Total RNA from primary tumor samples was isolated using the RNeasy Mini Kit (Qiagen) or Monarch Total RNA Miniprep kit (New England Biolabs GmbH). RNAseq was performed on retinoblastoma parental cell lines and models. Models were preincubated with/without 0.5 µg/ml doxycycline for 48 h before analysis. Total RNA from cells was prepared according to manufacturer’s instructions using RNeasy mini kit (Qiagen) with a separate DNAse I (Qiagen) digestion step. Whole-transcriptome sequencing on parental cell lines and tumor samples was performed by CeGat (Tübingen, Germany) using the SMART-Seq Stranded kit (Takara Bio Europe) for library preparation and NovaSeq 6000, 2x100bp (Illumina) for sequencing. For cell models, RNA amount was determined by Qubit fluorometer (Life Technologies), RNA quality was assessed by TapeStation 4200 with RNA ScreenTapes (Agilent) and the Lexogen QuantSeq 3′ mRNA-Seq Library Prep Kit FWD (Lexogen) was used to produce the library. Library quality were assessed on the TapeStation 4200 (High Sensitivity ScreenTapes, Agilent) and concentrations were measured using the Qubit system. RNA sequencing of *MYCN*-knockdown models were sequenced using either the Illumina NextSeq 2000 System (P2 reagents, 76 cycles, single-read, dual indices) or MiSeq using MiSeq Reagent Kit v3 (76 cycles, single-read, dual indices) using 3 biological replicates for each model and condition.

### DNA methylation data processing and consensus clustering

Two retinoblastomas that were previously profiled (450k) within the INFORM study^54–57^ to identify molecular targets for therapeutic options were reused for our analyses (Supplementary Data 1). Datasets generated on 450k and EPIC arrays were reduced to the 450 K common probes and processed using *minfi* (version 1.36), then normalized using *minfi*’s funnorm function. Batch correction was conducted for each array type using *comBat* (from sva version 3.42). The resulting methylation levels were discretized into 3 classes: unmethylated (levels close to 0), methylated (levels close to 1), and unknown or semi-methylated (remainder). Exact cut-off thresholds were determined by fitting a 3-component mixture model of beta distributions to the histogram of methylation levels and identifying points of equal probability density, as described previously^27^. Probes were then filtered to remove probes without information content, retaining only probes that showed the “methylated” or “unmethylated” state in at least 10 different samples. Consensus clustering was performed on the reduced discretized sample-probe matrix by applying different clustering algorithms from the Python scikit-learn package (K-means, affinity propagation, agglomerative or hierarchical with single, average and complete linkage and cosine, Euclidean and Manhattan distance; spectral clustering; DBSCAN; MeanShift) in combination with different preprocessing methods (robust scaling, normalization and principal component analysis or UMAP with 1 to 4 dimensions) and different output cluster numbers (2 to 6). From the resulting 8857 successful individual clusterings, those with a silhouette score of zero or less were discarded. Remaining clusterings were used to define a similarity matrix between samples, describing in how many clusterings two samples were assigned to the same cluster. The matrix was transformed into a similarity graph, such that each sample is a node and weighted edges between nodes indicate relative similarity (between 0 and 1; proportion of clusterings for which the 2 samples are in the same cluster). As a last step, edges with a weight <0.5 were removed resulting in 3 separated components defining the final 3 clusters.

### Differential methylation analysis

Retinoblastoma subtype-specific clustering was carried out using the published 8-probe classifier^12^, then confirmed using a modified 8-probe classifier supplemented with EPIC array probe cg04786667, which is closest to 450 array probe cg12750745 that is absent from the EPIC array. Hierarchical clustering (*dplyr* R package) was performed for the 8-probe classifiers with the parameters (distance: Manhattan, linkage: Ward.D2). Heatmaps were produced using the *gplots* R package. Differential methylation in retinoblastoma clusters was defined by differentially methylated probe sets for the batch-corrected (C*omBat*) whole-genome methylation data from tumors (Welch t-test with Benjamini–Hochberg false discovery rate adjustment; cut-off: β value mean difference of at least 0.2, adjusted *p* < 0.001). Cell lines were matched to cluster methylation profiles by extending the matrix for tumor methylation with β values from cell lines. Differential methylation in specified genomic regions (super-enhancers, CpG islands, etc) utilized the Bioconductor *regioneR* package on the usegalaxy.org public server^64^. CpG island coordinates were retrieved from the UCSC server (2020 update, http://hgdownload.cse.ucsc.edu/goldenpath/hg19/database/cpgIslandExt.txt.gz;). BED files corresponding to the enhancers identified in developing retinoblastoma and retina^30^ (GSE86981) were extracted from the SEDb2.0 database^31^. The Homer script, *findMotifs.pl* (http://homer.ucsd.edu/homer/, v4.10.3), was used to compare 50-bp sequences (containing known motifs) around CpGs (51 bp) that were differentially methylated with a strand-specific input.

### RNA sequencing data analysis

Differential gene expression was analyzed among the 52 retinoblastomas profiled by RNA sequencing. RNA sequencing reads were preprocessed and aligned to the *H. sapiens* normal transcriptome construct (Ensembl v96 release) and relative transcript abundance was quantified for each retinoblastoma using *kallisto* (-k 31 parameter, -b 100 parameter)^65^. To identify differential gene expression, *p-*values were computed using *sleuth* (Wald test), then used to generate false discovery rates (*q* values, using the *qvalue* package) that were adjusted for multiple comparisons using the Storey-Tibshirani method (bootstrap method to calculate π0 values)^66^. *p*- or *q*-values were combined using Edgington’s^67^ method and *metap* package. Box plot visualization for intertumor comparisons of differential expression data from the matrix created by *kallisto* required batch correction, which was performed using *removeBatchEffect* from *limma* package (Bioconductor) using *log1p*-*transformed tpm values*. Differential expression in cell lines and models was analyzed using *DESeq2*-based Quantseq 2.3.6 FWD pipeline and Quantseq DE 1.4.0 pipeline on the BlueBee® Genomics Platform (Lexogen). Differentially expressed genes were ranked in cell models and tumors using π-values^68^. Gene sets from MSigDB^69^ were used for GSEA with parameter settings (statistic: weighted, number of permutations: 1000, minimal gene set size: 10, maximal gene set size: 500 or 5000 for customized gene sets). Spearman coefficients for correlation were visualized with *corrplot*. Hierarchical clustering of RNA expression data was performed using the *dplyr* R package with the parameter settings (distance: Euclidean, linkage: Ward.D2). Heatmaps were produced using *gplots* R package. Higher-level chromosome rearrangements (translocations, duplications) were analyzed using the *Arriba* pipeline for RB_52 and RB_8 (RNA-STAR settings in Supplementary Data 2).

### Bioinformatic analyses

Chromosomal copy number variations (CNVs) were identified for the 61 samples by calling CNVs from the whole-genome methylation datasets for each sample using the Bioconductor *conumee* package^70^. Whole-exome sequencing data were analyzed using Varlociraptor v3.6.3 (https://github.com/snakemake-workflows/dna-seq-varlociraptor). Consensus clustering was performed using the R package *ConsensusClusterPlus* (settings: maxK = 4, reps = 1000, pItem = 0.8, pFeature = 1, clusterAlg = “hc”, distance = “Pearson”). Correlations between DNA methylation and gene expression were assessed using the Pearson correlation coefficient (cutoff: variance for β-values ≥ 1 10^−4^, Bonferroni-Hochberg-adjusted *p* < 0.05, R ≥ 0.4) and the batch-corrected matrix for normalized expression values of the corresponding genes. If multiple CpGs in a gene were differentially methylated, the Pearson correlation coefficient was calculated for each CpG-gene pair. Enrichment of expression or methylation gene signatures were analyzed using the *clusterProfiler* Bioconductor package^71^ with parameter settings (ontology: ALL, *p*-value cutoff: 0.05, method of *p*-value adjustment: Benjamini-Yekutieli, adjusted *p*-value cutoff: 0.25, minimal gene set size: 10, maximal gene set size: 500). Methylation probe mapping utilized RefSeq gene features. Venn diagrams for gene expression were generated using the *VennDiagram* and *BioVenn* R packages^72^. Box and volcano plots for gene expression and methylation β-value visualizations were generated using the *ggplot2* and *reshape2* R packages. Bioinformatic analyses were conducted using the R statistical package, v4.2.2 (2022) and Bioconductor libraries v3.16 (R Core Team, 2020). *P* values were adjusted using the Benjamini–Hochberg method^73^ for multiple comparisons, and unless otherwise stated, considered statistically significant if *p* < 0.05.

### Statistics and reproducibility

Data were expressed as mean ± SD or mean ± SE. Unless otherwise stated, all statistical tests are two-sided. Unless otherwise stated, *p* < 0.05 was regarded as statistically significant. Every experiment was repeated three times independently.

### Reporting summary

Further information on research design is available in the Nature Portfolio Reporting Summary linked to this article.

## Supplementary information

Supplementary Information

Description of Additional Supplementary Files

Supplementary Data 1-2

Supplementary Data 3-5

Supplementary Data 6-16

Supplementary Data 17-24

Supplementary Data 25-29

Supplementary Data 30

Reporting summary

## Data Availability

Raw DNA methylation data and RNA sequencing data have been deposited in GEO under study accession no. GSE267015 and GSE268136. GSE59983^13^, GSE58785^12^, and GSE86981^30^ datasets were used for the analyses. The source data for graphs and charts are available in Supplementary Data 30. All other data (raw WES data and the code for consensus clustering) will be provided upon reasonable request.
