# Author Correction: A MYCN-driven de-differentiation profile identifies a subgroup of aggressive retinoblastoma

**DOI:** 10.1038/s42003-024-06830-1

**Published:** 2024-09-11

**Authors:** Tatsiana Ryl, Elena Afanasyeva, Till Hartmann, Melanie Schwermer, Markus Schneider, Christopher Schröder, Maren Wagemanns, Arthur Bister, Deniz Kanber, Laura Steenpass, Kathrin Schramm, Barbara Jones, David T. W. Jones, Eva Biewald, Kathy Astrahantseff, Helmut Hanenberg, Sven Rahmann, Dietmar R. Lohmann, Alexander Schramm, Petra Ketteler

**Affiliations:** 1 Department of Pediatric Hematology and Oncology, University Hospital Essen, Essen, Germany; 2 Algorithms for Reproducible Bioinformatics, Genome Informatics, Institute of Human Genetics, University Hospital Essen, University of Duisburg-Essen, Essen, Germany; 3 Institute of Human Genetics, University Hospital Essen, University Duisburg Essen, Essen, Germany; 4 Human and Animal Cell Lines, Leibniz Institute DSMZ German Collection of Microorganisms and Cell Cultures, Braunschweig, Germany; 5 Division of Pediatric Glioma Research, Hopp Children’s Cancer Center (KiTZ), Heidelberg, Germany; 6 National Center for Tumor Diseases (NCT), NCT Heidelberg, a partnership between DKFZ and Heidelberg University Hospital, Heidelberg, Germany; 7 German Cancer Research Center (DKFZ), Heidelberg, Germany; 8 Department of Pediatric Hematology and Oncology, Heidelberg University Hospital, University of Heidelberg, Heidelberg, Germany; 9 Department of Ophthalmology, Medical Faculty, University of Duisburg-Essen, Essen, Germany; 10 Department of Pediatric Oncology and Hematology, Charité—University Medicine Berlin, Berlin, Germany; 11 Algorithmic Bioinformatics, Center for Bioinformatics Saar and Saarland University, Saarland Informatics Campus, Saarbrücken, Germany; 12 Laboratory for Molecular Oncology, Department of Medical Oncology, West German Cancer Center, University Hospital Essen, University of Duisburg-Essen, Essen, Germany

**Keywords:** Paediatric cancer, Cancer epigenetics, Eye cancer

**Correction to:** *Communications Biology* 10.1038/s42003-024-06596-6, published online 30 July 2024

In this article, the author name Hanenberg was incorrectly written as Hannenberg. The original article has been corrected.

