# Supplementary material for: A MYCN-driven de-differentiation profile identifies a subgroup of aggressive retinoblastoma
Source: Commun Biol. 2024 Jul 30;7:919. doi: 10.1038/s42003-024-06596-6 (PMC11289481; doi:10.1038/s42003-024-06596-6)
Supplement: Supplementary file 1 — Supplementary Information [file 42003_2024_6596_MOESM1_ESM.pdf]

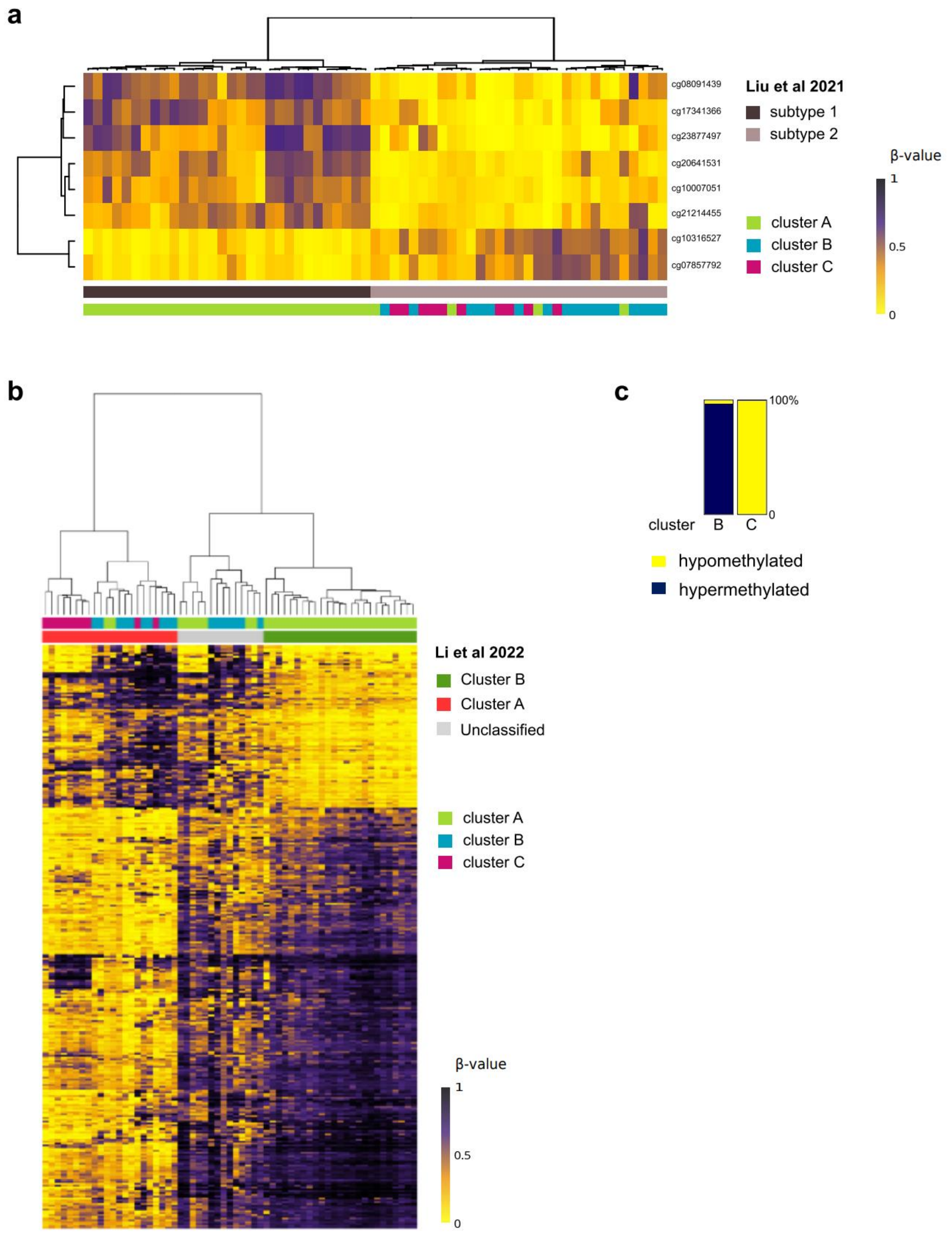

Figure S1. DNA methylation characteristics of retinoblastoma clusters. Heatmaps show results of hierarchical clustering for 61 retinoblastomas using DNA methylation β-values from 8 probes (850K array) in the Liu *et al*. classifier[12] (**a**) and from the panel of 320 probes discriminating between Clusters A and B in Li *et al*[28] (**b**). **c,** Proportion of hypomethylated (yellow) and hypermethylated (blue) genomic locations in clusters C versus A|B and in clusters B versus A|C.

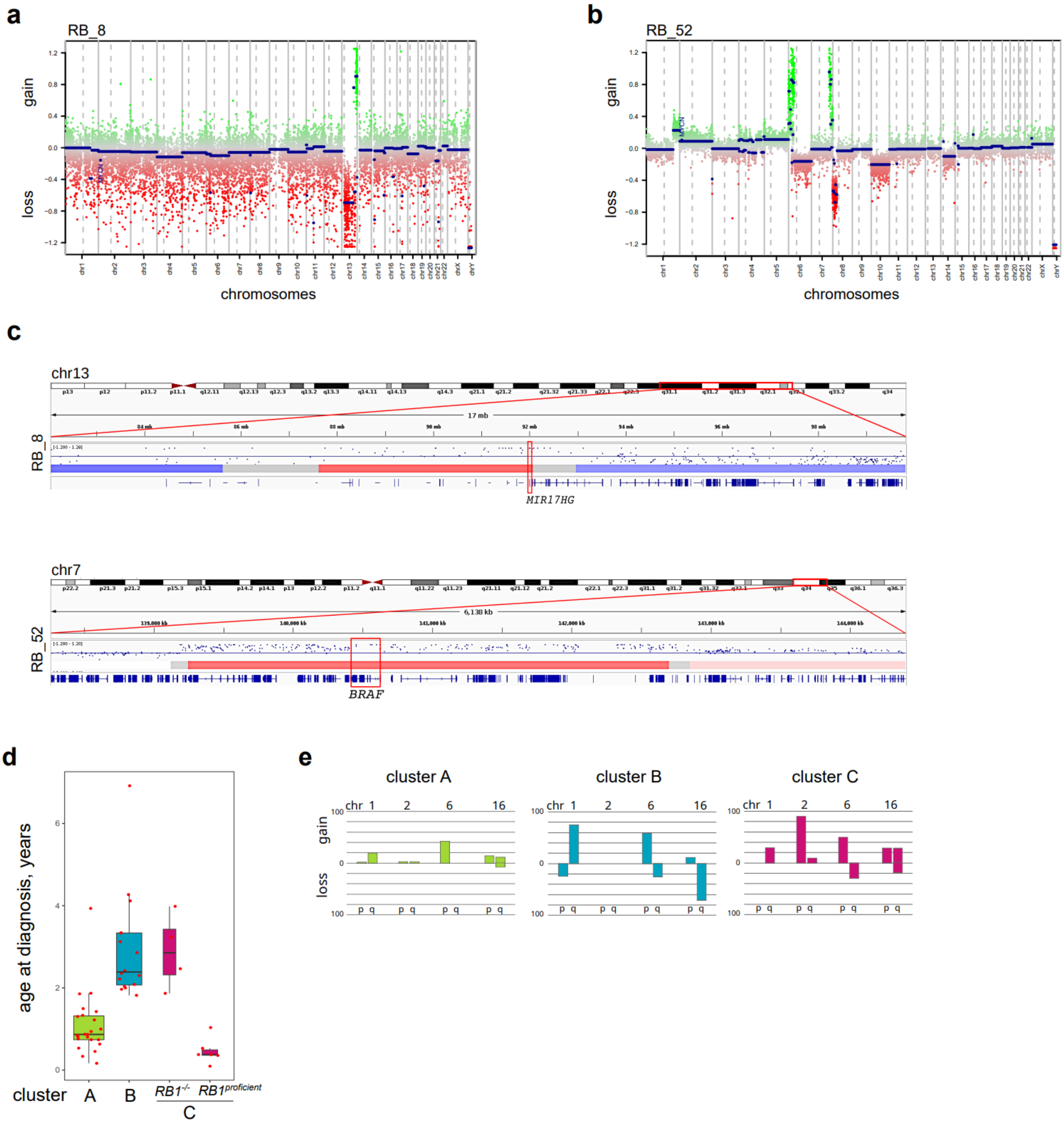

a
RB_8
gain
loss
chromosomes
b
RB_52
chromosomes
c
chr13
RB_8
MIR17HG
chr7
RB_52
BRAF
d
age at diagnosis, years
cluster A B RB1-/- RB1proficient
C
e
cluster A
cluster B
cluster C

8

Figure S2. Clinical, epigenetic and genetic features of retinoblastoma clusters. Copy number variation plots based on DNA methylation array data in RB_8 (**a**) and RB_52 tumors (**b**). The log2 copy number ratio is shown on the y-axis; for every chromosome, the p-arm (left) and q-arm (right) are separated by a dotted line. Gains or amplification (green) were calculated as positive values placed above the baseline, and losses or deletions (red) as negative values under the baseline. **c**, Segmented copy number plots demonstrating gains (red) at the loci *MIR17HG* in RB_08 (upper panel) and *BRAF* in RB_52 (lower panel) tumors. **d,** Box plot demonstrating age at diagnosis in the retinoblastoma clusters; cluster C is subdivided according to *RB1* status (RB1-proficient (n=6); $RB1^{-/-}$ (n=4)). Each dot corresponds to one tumor. Boxes show the first to third quartile with median. Whiskers extend from the hinge to ±1.5 times the interquartile range or the highest/lowest value. **e,** Somatic copy number alteration frequency for chromosomes 1, 2, 6 and 16 in clusters A, B and C.

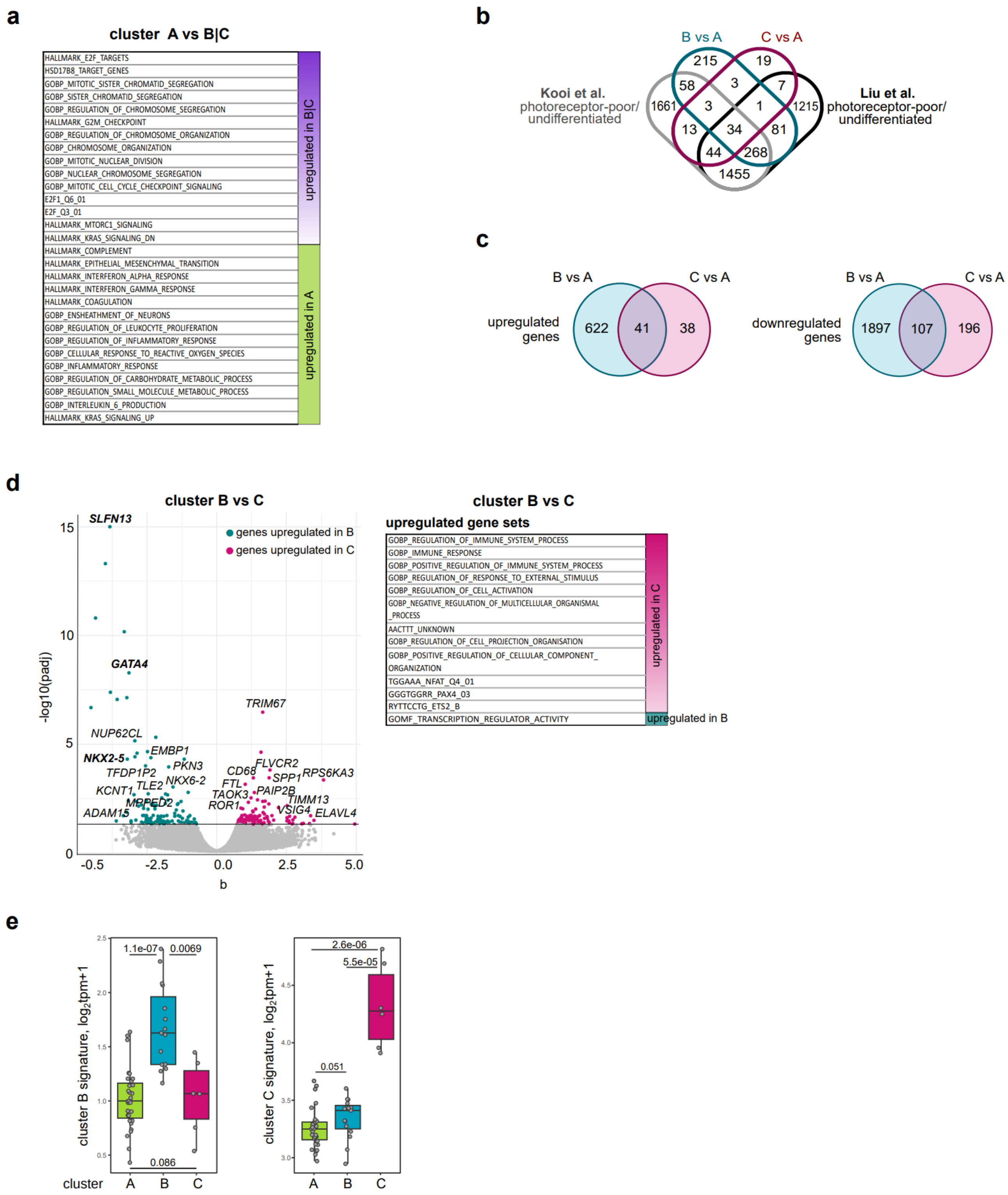

Figure S3. Gene expression analysis of retinoblastoma clusters. **a,** Table showing most significant gene sets from three datasets: H: hallmark gene sets, C3: regulatory target gene sets, C5: ontology gene sets (MsigDB) in B|C vs. A. **b,** Venn diagram showing the overlap between genes upregulated in cluster B vs. cluster A, upregulated in cluster C vs. cluster A and genes representative of photoreceptor-poor, undifferentiated retinoblastoma[12, 13]. **c,**

Venn diagram showing the overlap between upregulated (left) and downregulated genes (right) in cluster B vs. cluster A and cluster C vs. cluster A (complete output, **Supplementary Data 10**). **d,** Volcano plot of differentially expressed transcripts between clusters B and C (left). Up- and downregulated transcripts are plotted in magenta and turquoise, respectively. Key transcripts are plotted with corresponding annotated gene symbols (**Supplementary Data 13**). Tables (right) showing most significant gene sets from three datasets: H: hallmark gene sets, C3: regulatory target gene sets, C5: ontology gene sets (MsigDB). **e,** Box plots showing distribution of expression scores of genes from cluster B (524 transcripts) and cluster C (117 transcripts) signatures (**Supplementary Data 15, 16**) in 52 retinoblastomas. Boxes show the first to third quartile with median. Whiskers extend from the hinge to ±1.5 times the interquartile range.

a

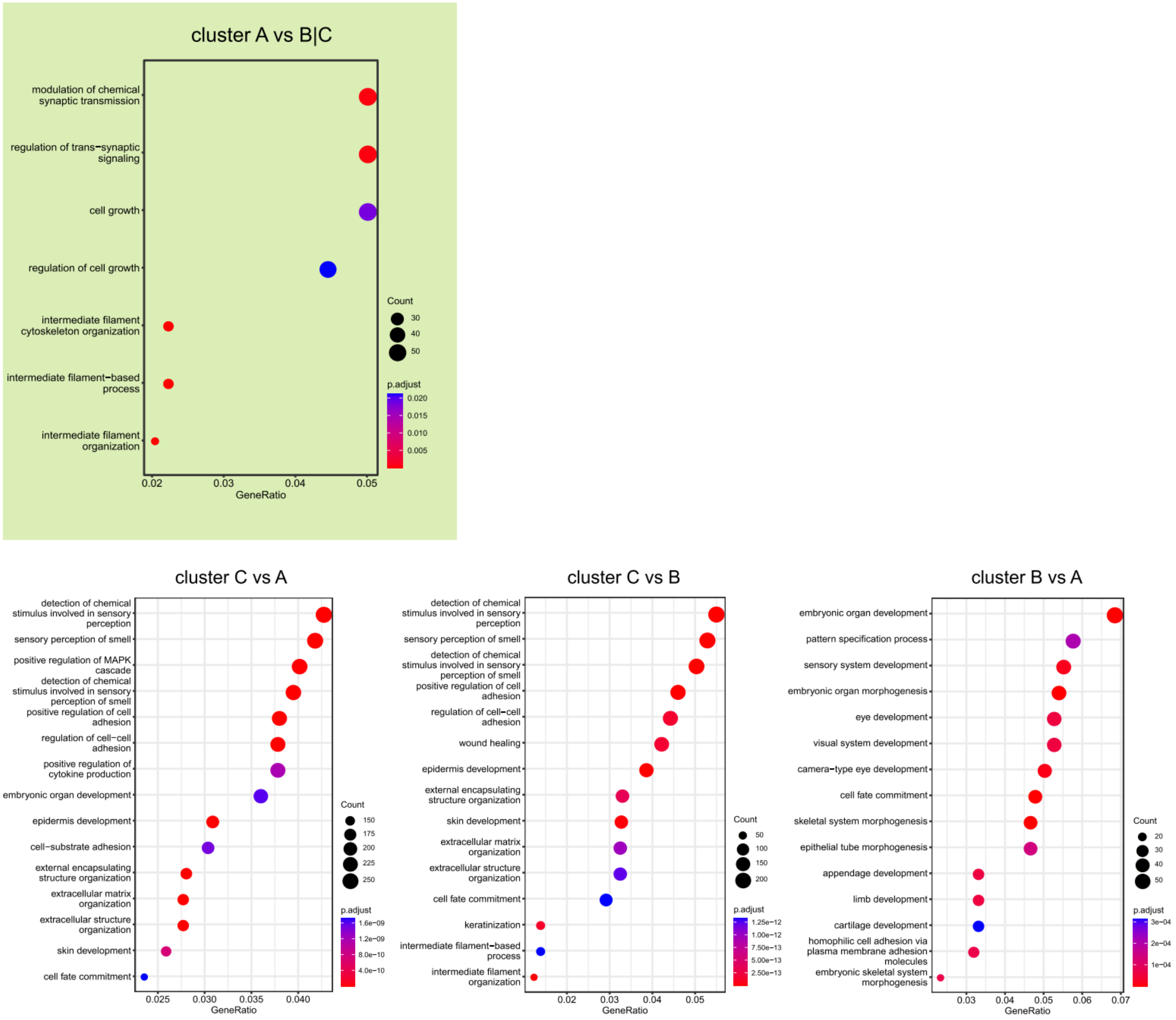
cluster A vs B|C
cluster C vs A
cluster C vs B
cluster B vs A
GeneRatio
Count
p.adjust

b

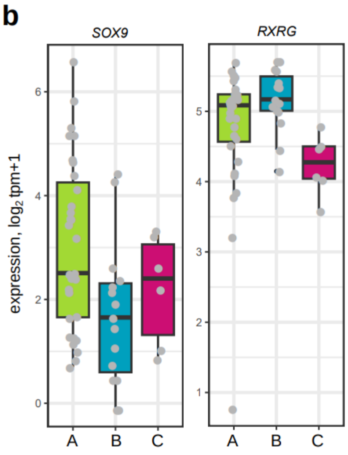
SOX9
RXRG
expression, log2 tpm+1
A
B
C

Figure S4. Integrated DNA methylation and gene expression analyses of retinoblastoma clusters. **a,** GO enrichment analysis showing biological processes overrepresented by the genes matching to differentially methylated CpGs in four comparisons, A vs B|C, C vs A, C vs B and B vs A. Dot size and color represent the number of genes and enrichment significance, respectively. X-axis indicates the gene enrichment ratio (GeneRatio) of a biological process GO term. **b,** Box plots showing aggregated log1p-transformed transcript tpm (transcripts per kilobase million) values of *SOX9* and *RXRG* in 52 retinoblastomas. Boxes show the first to third quartile with median. Whiskers extend from the hinge to ±1.5 times the interquartile range.

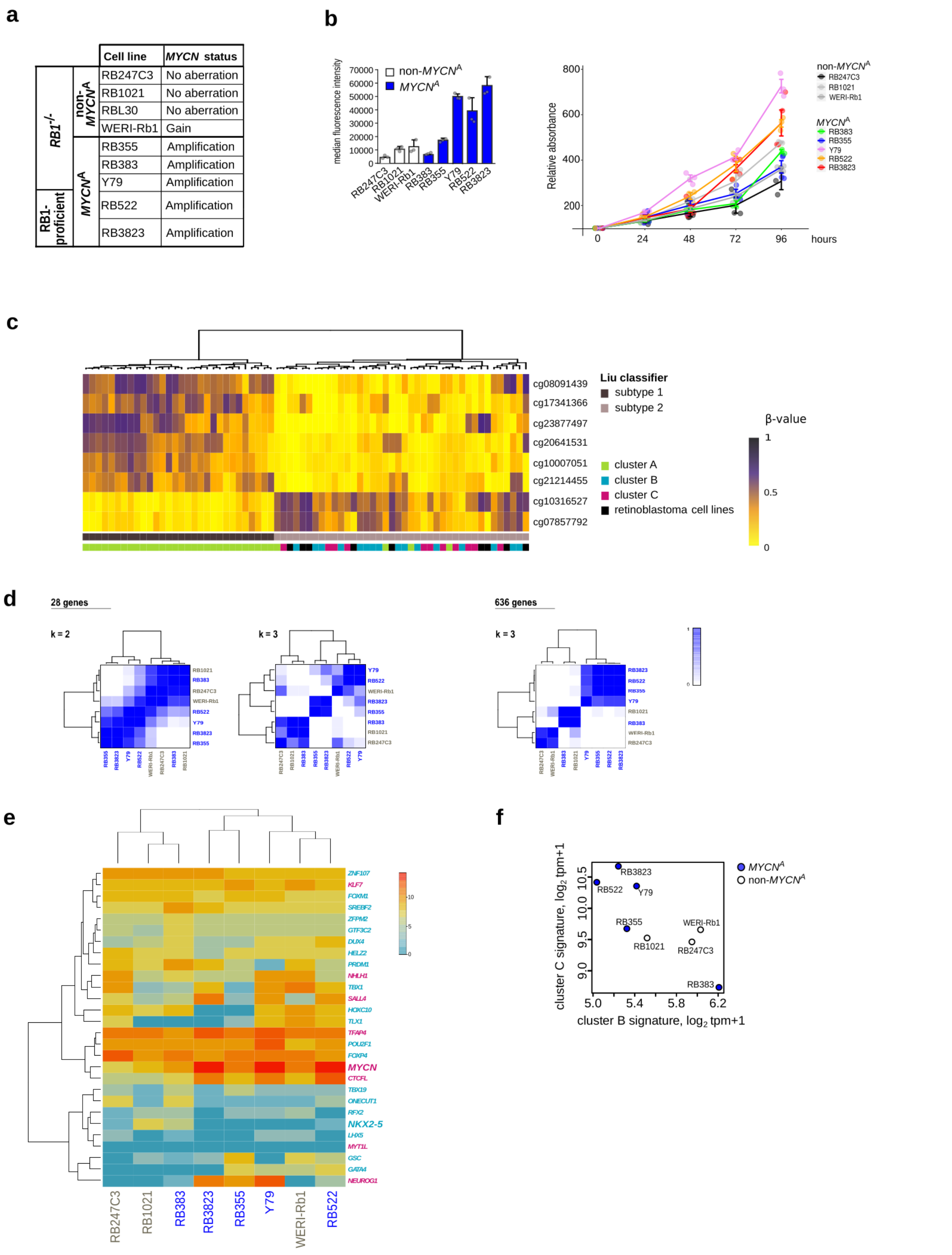

Figure S5. Proliferation, DNA methylation and gene expression analyses of 8 retinoblastoma cell lines. **a,** Summary information on retinoblastoma cell lines used in the

study with the statuses of *RB1* and *MYCN*. **b,** MYCN protein expression (left) and cell viability (right) in 8 retinoblastoma cell lines. MYCN expression was determined by fluorescence activated cell sorting (FACS); and cell viability was measured by MTT-assay. Data in the figure are mean + SD (protein expression) and mean ± SE (cell viability) from 3 experiments with dots representing individual values. MTT values were normalized to the control value and presented as fold change with respect to control at different time intervals. **c,** Heatmap showing results of hierarchical cluster analysis on 61 tumors and 9 retinoblastoma cell lines using DNA methylation ß-values of 8 probes (850K Array) from the classifier by Liu et al[12]. **d,** Consensus clustering matrices for 2 (k = 2) and 3 (k = 3) partitions obtained from expression of 28 cluster C- and cluster B-specific TFs and 636 cluster C- and cluster B-specific transcripts. Consensus values ranging from 1 (the darkest blue, indicates cell lines that always co-clustered) to 0 (white, indicates cell lines that never clustered together) are indicated. **e,** Hierarchical clustering of mRNA expression (lower panel) showing cluster C- and cluster B-specific TFs in 8 retinoblastoma cell lines. **f,** Scatter plots showing the relationship between expression of genes from cluster C (116 transcripts, x-axis) and cluster B (520 transcripts, y-axis) signatures in 8 retinoblastoma cell lines. The average of log1p-transformed tpm values for a gene set was used as a signature score of each tumor.

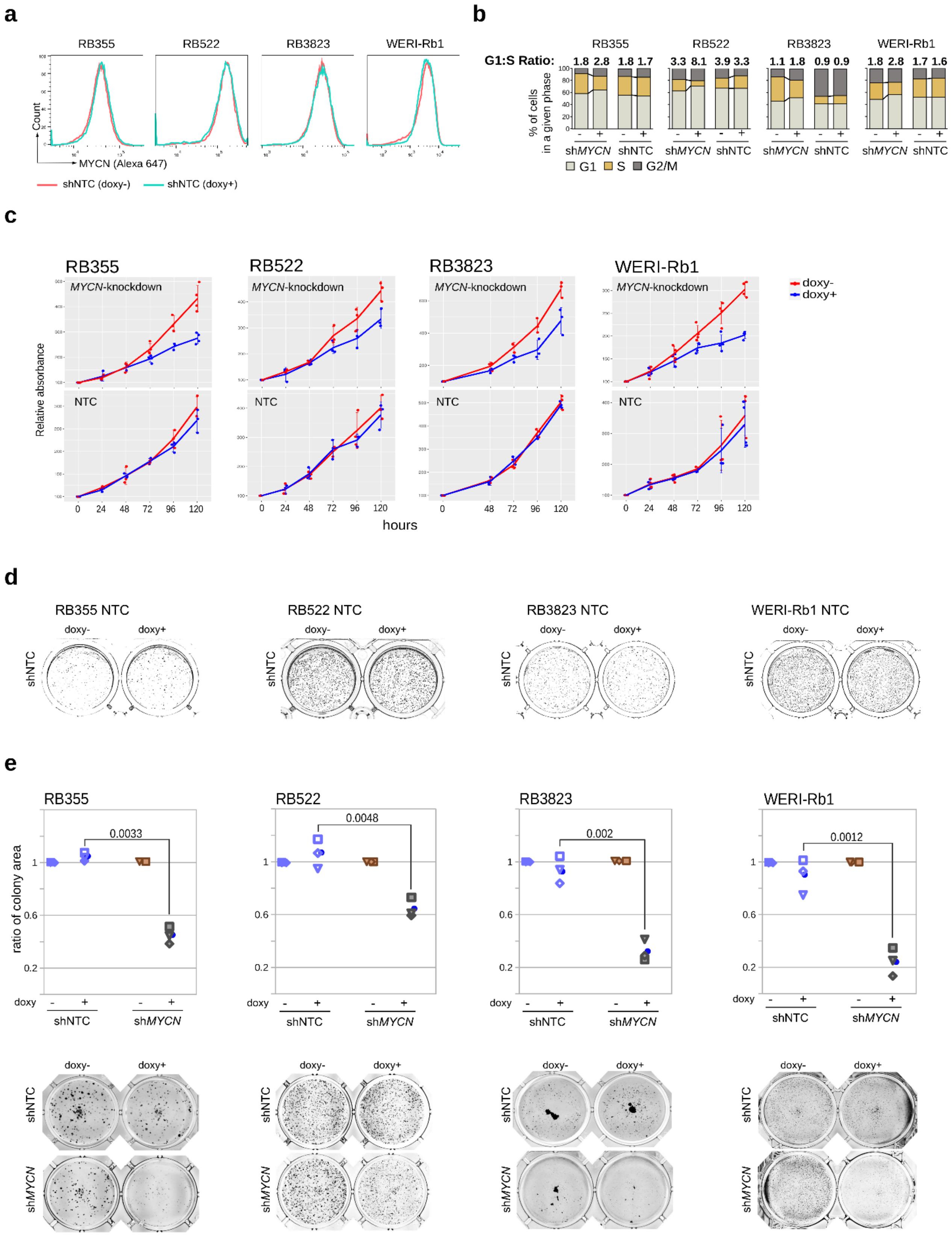

a
RB355
RB522
RB3823
WERI-Rb1
Count
MYCN (Alexa 647)
shNTC (doxy-)
shNTC (doxy+)
b
G1:S Ratio:
% of cells in a given phase
shMYCN
shNTC
G1
S
G2/M
c
MYCN-knockdown
NTC
Relative absorbance
hours
doxy-
doxy+
d
RB355 NTC
RB522 NTC
RB3823 NTC
WERI-Rb1 NTC
e
ratio of colony area
0.0033
0.0048
0.002
0.0012
doxy

Figure S6. Proliferation and gene expression analyses of 4 *MYCN*-knockdown retinoblastoma cell lines. **a,** Flow cytometry analysis of MYCN protein expression in RB355, RB522, RB3823 and WERI-Rb1 cell lines transduced with doxycycline-inducible lentiviral shNTC expression vector. MYCN protein was measured 48h after adding doxycycline to the media. Data are from 3 independent experiments. **b,** Cell cycle analysis in four *MYCN*-knockdown cells. Cells expressing sh*MYCN* and shNTC were incubated with doxycycline or vehicle for 96h. DNA synthesis (S-phase) determined by EdU incorporation and the whole DNA content by DAPI staining. The results represent three experiments. **c,** Cell viability in RB355, RB522, RB3823 and WERI-Rb1 cell lines transduced with either doxycycline-inducible sh*MYCN* or with shNTC lentiviral expression systems as measured by MTT-assay. To quantify the metabolic viability, MTT values. Values were normalized to the control value and presented as fold change with respect to control at different time intervals. Data are from 3 independent experiments with dots representing individual values. **d,** Representative images from shNTC-expressing non-treated and doxycycline-treated four cell lines in soft agar. **e,** Clonogenicity assay in *MYCN*-knockdown retinoblastoma cells. Plots (upper panel) show ratios of total colony areas in shNTC-expressing (doxycycline-treated cells vs non-treated cells) and sh*MYCN*-expressing (doxycycline-treated cells vs non-treated cells) retinoblastoma cell lines from three independent experiments. Data are from 3 independent experiments, with average values (blue bots) indicated and *p*-values calculated with Welch t-test reported. Representative images (lower panel) from four shNTC-expressing and sh*MYCN*-expressing non-treated and doxycycline-treated cell lines in clonogenicity assay.

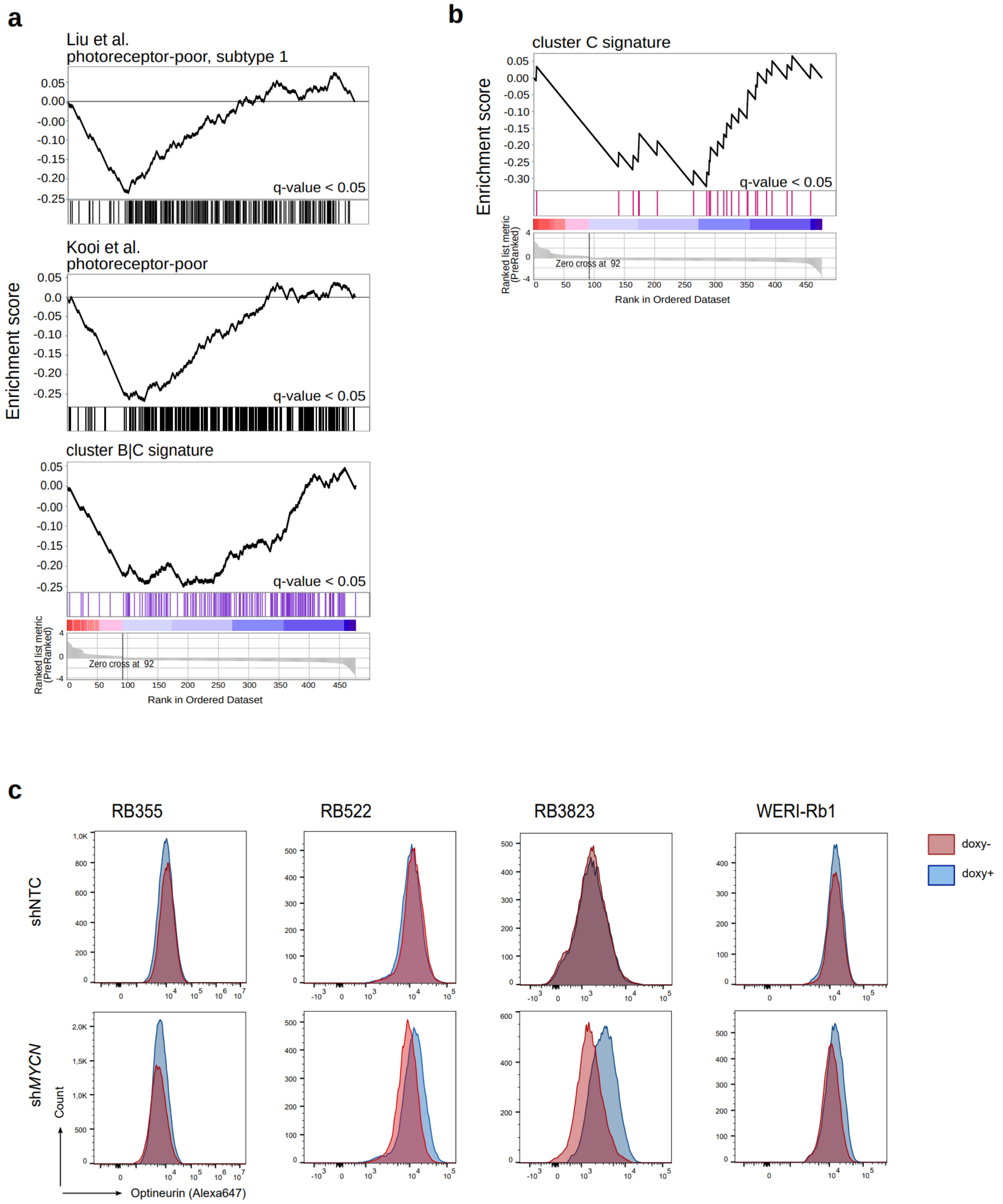

Figure S7. Proliferation and gene expression analyses of 4 *MYCN*-knockdown retinoblastoma cell lines. GSEA showing genes positively expressed in photoreceptor-poor, undifferentiated retinoblastoma[12, 13] (**a**) and cluster C (**b**) in the *sleuth*-modeled *MYCN*-knockdown expression pattern. Y-axis indicates enrichment score (ES). X-axis shows pathway genes. The dual-colored band represents the degree of correlation in the expression of these genes with *MYCN*-knockdown (red, *MYCN*-knockdown; blue, control).

c, Representative histograms of geometric mean fluorescence intensity (MFI) for OPTN in four *MYCN*-knockdown and shNTC-expressing non-treated and doxycycline-treated cell lines.

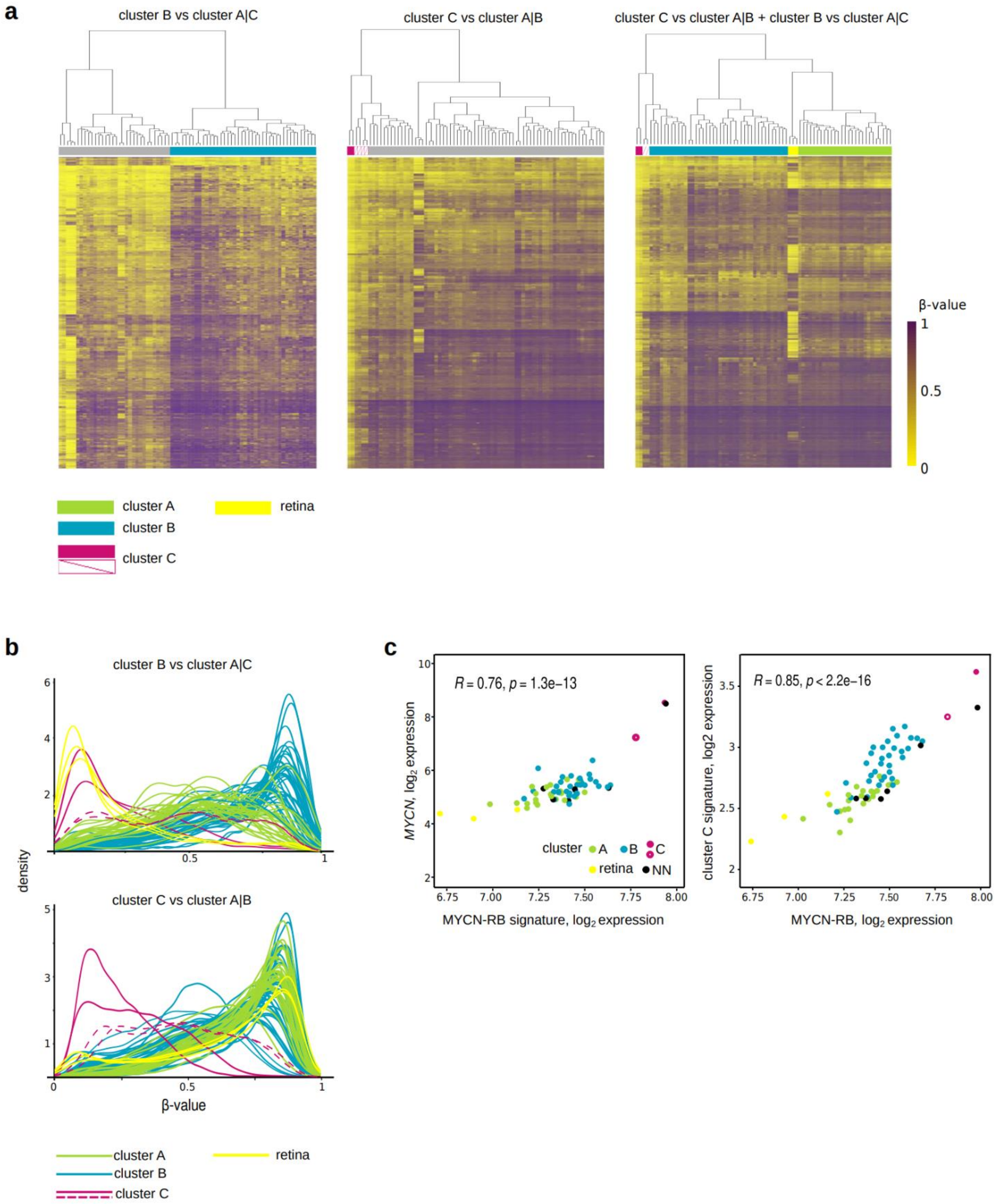

Figure S8. Validation of cluster C DNA methylation and expression signatures. **a,** Heatmap showing results of hierarchical cluster analysis on GSE58783 cohort[12] (n = 74) using DNA methylation ß-values of cluster B vs A|C specific CpGs, cluster C vs A|B specific CpGs and the combined signature of cluster B vs A|C and cluster C vs A|B CpGs. **b,** Density plots for DNA methylation levels (ß-values) of differentially methylated probes in cluster B

vs. A|C (upper panel) and cluster C vs. A|B (lower panel) in GSE58783 cohort (n = 74). **c,** Scatter plots showing the relationship between expression of *MYCN* (y-axis, left panel), or cluster C signature (right panel, y-axis), and MYCN-RB signature (x-axes) in GSE58780 cohort[12] (n = 66). The average of log-transformed expression values for a gene set was used as a signature score of each tumor. Pearson (R) coefficients with corresponding *p*-values are indicated for each comparison.
