# Supplementary material for: A MYCN-driven de-differentiation profile identifies a subgroup of aggressive retinoblastoma
Source: Commun Biol. 2024 Jul 30;7:919. doi: 10.1038/s42003-024-06596-6 (PMC11289481; doi:10.1038/s42003-024-06596-6)
Supplement: Supplementary file 2 — Description of Additional Supplementary Files [file 42003_2024_6596_MOESM2_ESM.pdf]

Description of Additional Supplementary Files

File name: Supplementary Data 1-2
Description: Retinoblastoma cohort metadata.

File name: Supplementary Data 3-5
Description: Specific rearrangements and mutations and detected in the retinoblastoma cohort.

File name: Supplementary Data 6-16
Description: Gene expression analysis of retinoblastoma clusters.

File name: Supplementary Data 17-24
Description: Integrated DNA methylation and gene expression data for 52 retinoblastomas.

File name: Supplementary Data 25-29
Description: Gene expression analysis of MYCN-knockdown retinoblastoma cell models.

File name: Supplementary Data 30
Description: Source data behind the graphs and charts in the paper
